# A systematic review on the performance of fracture risk assessment tools: FRAX, DeFRA, FRA-HS

**DOI:** 10.1007/s40618-023-02082-8

**Published:** 2023-04-09

**Authors:** G. Adami, A. Biffi, G. Porcu, R. Ronco, R. Alvaro, R. Bogini, A. P. Caputi, L. Cianferotti, B. Frediani, D. Gatti, S. Gonnelli, G. Iolascon, A. Lenzi, S. Leone, S. Migliaccio, T. Nicoletti, M. Paoletta, A. Pennini, E. Piccirilli, U. Tarantino, M. L. Brandi, G. Corrao, M. Rossini, R. Michieli

**Affiliations:** 1 Rheumatology Unit, University of Verona, Verona, Italy; 2 Department of Statistics and Quantitative Methods, National Centre for Healthcare Research and Pharmacoepidemiology, University of Milano-Bicocca, Milan, Italy; 3 Unit of Biostatistics, Epidemiology, and Public Health, Department of Statistics and Quantitative Methods, University of Milano-Bicocca, Milan, Italy; 4 Department of Biomedicine and Prevention, University of Rome Tor Vergata, Rome, Italy; 5 Local Health Unit (USL) Umbria, Perugia, Italy; 6 Department of Pharmacology, School of Medicine, University of Messina, Messina, Italy; 7 Italian Bone Disease Research Foundation (FIRMO), Florence, Italy; 8 Department of Medicine, Surgery and Neurosciences, Rheumatology Unit, University of Siena, Azienda Ospedaliero-Universitaria Senese, Siena, Italy; 9 Department of Medicine, Surgery and Neuroscience, Policlinico Le Scotte, University of Siena, Siena, Italy; 10 Department of Medical and Surgical Specialties and Dentistry, University of Campania “Luigi Vanvitelli”, Naples, Italy; 11 Department of Experimental Medicine, Sapienza University of Rome, Viale del Policlinico, Rome, Italy; 12 AMICI Onlus, Associazione Nazionale per le Malattie Infiammatorie Croniche dell’Intestino, Milan, Italy; 13 Department of Movement, Human and Health Sciences, Foro Italico University, Rome, Italy; 14 Coordinamento Nazionale delle Associazioni dei Malati Cronici e rari di Cittadinanzattiva, CnAMC, Rome, Italy; 15 Department of Clinical Sciences and Translational Medicine, University of Rome “Tor Vergata”, Rome, Italy; 16 Department of Orthopedics and Traumatology, “Policlinico Tor Vergata” Foundation, Rome, Italy; 17 Italian Society of General Medicine and Primary Care (SIMG), Florence, Italy

**Keywords:** Fracture risk assessment, Fragility fracture, Secondary prevention, Systematic review

## Abstract

**Purpose:**

Preventing fragility fractures by treating osteoporosis may reduce disability and mortality worldwide. Algorithms combining clinical risk factors with bone mineral density have been developed to better estimate fracture risk and possible treatment thresholds. This systematic review supported panel members of the Italian Fragility Fracture Guidelines in recommending the use of best-performant tool. The clinical performance of the three most used fracture risk assessment tools (DeFRA, FRAX, and FRA-HS) was assessed in at-risk patients.

**Methods:**

PubMed, Embase, and Cochrane Library were searched till December 2020 for studies investigating risk assessment tools for predicting major osteoporotic or hip fractures in patients with osteoporosis or fragility fractures. Sensitivity (Sn), specificity (Sp), and areas under the curve (AUCs) were evaluated for all tools at different thresholds. Quality assessment was performed using the Quality Assessment of Diagnostic Accuracy Studies-2; certainty of evidence (CoE) was evaluated using the Grading of Recommendations Assessment, Development and Evaluation approach.

**Results:**

Forty-three articles were considered (40, 1, and 2 for FRAX, FRA-HS, and DeFRA, respectively), with the CoE ranging from very low to high quality. A reduction of Sn and increase of Sp for major osteoporotic fractures were observed among women and the entire population with cut-off augmentation. No significant differences were found on comparing FRAX to DeFRA in women (AUC 59–88% vs. 74%) and diabetics (AUC 73% vs. 89%). FRAX demonstrated non-significantly better discriminatory power than FRA-HS among men.

**Conclusion:**

The task force formulated appropriate recommendations on the use of any fracture risk assessment tools in patients with or at risk of fragility fractures, since no statistically significant differences emerged across different prediction tools.

**Supplementary Information:**

The online version contains supplementary material available at 10.1007/s40618-023-02082-8.

## Introduction

Osteoporosis is a chronic disease characterized by bone fragility, which leads to an increased risk of fractures [2]. As fragility fractures are a leading cause of disability and mortality worldwide, osteoporosis treatment should primarily aim at preventing fractures [1].

Low bone mineral density (BMD) is a major determinant of risk; it has been demonstrated that an increase in BMD is associated with fracture risk reduction in a quasi-linear manner [3]. However, BMD combined with clinical risk factors predicts fracture risk better than BMD alone [4]; these include: comorbidities, treatment with glucocorticoids, or a history of previous fractures. These factors are independent predictors of fracture and are associated with deterioration of bone quality [2]. Algorithms that combine clinical risk factors with BMD have been developed to better estimate fracture risk and determine possible thresholds for treatment [5–7].

The most widely used algorithm is the Fracture Risk Assessment Tool (FRAX), which was originally developed in 2008 by the World Health Organization collaborating center of the University of Sheffield, UK [6]. In Italy, other FRAX-derived tools (DeFRA and FRA-HS) are widely used for calculating fracture risk. The DeFRA was developed in 2010 by the Italian Society for Osteoporosis, Mineral Metabolism, and Bone Diseases (SIOMMMS) and the Italian Society of Rheumatology (SIR) [5]. The FRA-HS was developed and published by the Italian Society of General Practitioners (SIMG) [8]. Both algorithms have been validated against FRAX in post-menopausal women with osteoporosis [8, 9]. DeFRA considers the following patients’ clinical and densitometric characteristics for fracture risk calculation: age, weight, height, number and site of prior fragility fracture, parental history of hip and clinical vertebral fractures, glucocorticoid intake (semi-quantitative variable), treatment with adjuvant hormone therapy for breast cancer, the presence of various comorbidities (including rheumatoid arthritis, multiple sclerosis, psoriatic arthritis, systemic lupus erythematosus, other connective tissue disease), calcium intake from diet and supplements, vitamin D intake, falls, exposure to sunlight and both lumbar spine and femoral neck BMD [5].

FRA-HS estimate the fracture risk upon these characteristics: age, sex, history of osteoporotic fractures (dichotomic variable), secondary osteoporosis (dichotomic variable), long-term use of corticosteroids (dichotomic variable, at least 180 defined daily dose within the year prior to assessment), rheumatoid arthritis diagnosis, body mass index, smoking (dichotomic variable), and alcohol abuse/alcohol-related diseases (dichotomic variable) [8].

The Italian National Institute of Health (*Istituto Superiore di Sanità*) recently published the Italian guidelines “Diagnosis, risk stratification and continuity of care of Fragility Fractures” [10]. In regard to risk stratification, the task force focused on the three most commonly used fracture risk assessment tools in Italy (DeFRA, FRAX, and FRA-HS). A systematic review was conducted for each of these tools with the aim of assessing their clinical performance in patients at risk of fractures; the review also aimed to accumulate all relevant literature for formulating evidence-based recommendations. Herein, we present the results of the systematic review and meta-analysis on the performance of fracture risk assessment tools in patients at risk of fracture. The present meta-analysis informed the guidelines of the Italian National Institute of Health on fragility fractures.

## Materials and methods

A systematic review was performed to support the panel members of the Italian Fragility Fracture Guidelines (published on the platform of the Italian National Institute of Health [11]) in formulating recommendations. In accordance with the GRADE-ADOLOPMENT methodology [12] and the standards elaborated by the Sistema Nazionale Linee Guida (SNLG) [13, 14], the multidisciplinary panel aimed to answer the following clinical question: “Which risk assessment tools are the most accurate in predicting the risk of fragility fractures in adults, including those without known osteoporosis or previous fragility fractures?”. The recommendations from the CG146 guideline of the National Institute for Clinical Excellence (NICE) (which assessed fragility fracture risk in patients with osteoporosis) were updated and adapted for this review.

### Inclusion and exclusion criteria

Observational studies were selected if they met the following criteria: (1) population: patients with osteoporosis or those who had experienced a fragility fracture, according to the diagnostic criteria for osteoporosis and the definition of fragility given by different studies’ authors. In the vast majority of studies osteoporosis was defined based on T-score levels, fragility fracture was defined as: any asymptomatic morphometric vertebral fractures and/or any clinical bone fracture resulting from a fall from standing height or less or for a low-energy trauma; (2) risk assessment tools: FRAX [15], DeFRA [16], and FRA-HS [17]; reference standard: risk threshold for major osteoporotic fractures (MOF) (3%, 5%, 10%, 20%, and 30%) and hip fractures (3% and 5%), either with or without the BMD criterion; (3) outcome: (i) primary outcome measures of sensitivity (Sn) (capacity to correctly detect the fracture risk) and specificity (Sp) (exclusively identified fracture-free patients) for the risk assessment tools (studies were required to have Sn and Sp values, an adequate 2 × 2 table, or adequate data for creating the 2 × 2 table). Moreover, (ii) secondary outcomes were the receiver operating characteristic curve and the area under the curve (AUC) for Sn and Sp and, to easier interpret their goodness of fit, values were expressed in percentages by multiplying per 100.

Studies were excluded if they: (i) were not published in the English language, (ii) did not report original findings (i.e., letters and case reports), (iii) did not identify patients affected by fragility fractures or osteoporosis, or (iv) did not consider the risk assessment tools of interest (FRAX, DeFRA, or FRA-HS).

### Data source and search strategy

PubMed, Embase, and the Cochrane Library were searched (between September 2011 and December 2020) by updating the search strategy of the NICE guidelines for the FRAX tool; a new search was conducted for the DeFRA and FRA-HS tools. Publications on the risk assessment tools were identified in patients with fragility fractures or osteoporosis. The systematic review was conducted according to the Preferred Reporting Items for Systematic Reviews and Meta-analyses (PRISMA) [18]; the statement has been provided in Supplemental Table S1. The search strategy (Supplemental Material, A) included specific keywords and/or corresponding Medical Subject Headings terms related to fragility fracture/osteoporosis AND risk assessment tools. The reference lists of the studies were checked and systematic reviews were identified during the search process.

### Study selection and data extraction

Three independent authors (AB, GP, and RR) screened the titles and abstracts based on the search strategy and then assessed the full text of potentially relevant studies. Discrepancies between readers were resolved in conference.

The following data were extracted for each included observational study: (i) first author, year, and country of publication; (ii) study setting; (iii) duration of study; (iv) type of population; (v) intervention; and (vi) outcome (Supplemental Material, B).

### Study quality

The methodological quality of the included studies was evaluated using the Quality Assessment of Diagnostic Accuracy Studies version 2 (QUADAS-2) checklist [19]. The QUADAS-2 assessment was structured in four key domains: patient selection, index test, reference standard, flow and timing (Supplemental Table S2).

### Quality of evidence

The quality of evidence for each primary outcome was assessed based on five dimensions (risk of bias, consistency of effect, imprecision, indirectness, and publication bias) using the GRADE approach [20]. If serious or very serious limitations were found for each of the 5 dimensions, the evidence was downgraded from “high quality” by 1 and 2 levels, respectively.

### Statistical analysis

The following operating characteristics were evaluated for analysis of the risk assessment tool: the Sn and Sp (at different thresholds) and the AUC. Specific thresholds were used to differentiate between individuals with or without the target condition. In this context, the development group of the NICE guidelines established risk thresholds for MOF (3%, 5%, 10%, 20%, and 30%) and hip fractures (3%, 5%, and 10%). A low Sn implied that the tool did not recognize a proportion of MOFs or hip fractures; conversely, a low Sp indicated that the tool could lead to false positive cases and overestimate the incidence of these fractures. Analyses were therefore performed when studies reported different cut-off values for the same risk assessment tool.

The Sn and Sp estimates were used to realize coupled forest plots with 95% confidence intervals (CIs) across studies (at various thresholds); RevMan V.5.4 (Nordic Cochrane Center) software was used for evaluation. The AUC was used to evaluate the overall diagnostic accuracy of each risk assessment tool. Diagnostic meta-analysis was conducted when 3 or more studies were available per threshold. This measure was also plotted on a graph using RStudio software version 1.4.1717. Heterogeneity or inconsistency among studies was visually inspected using the forest plots for MOF or hip fractures, both with and without BMD.

## Results

### Study selection

**Fig. 1:**
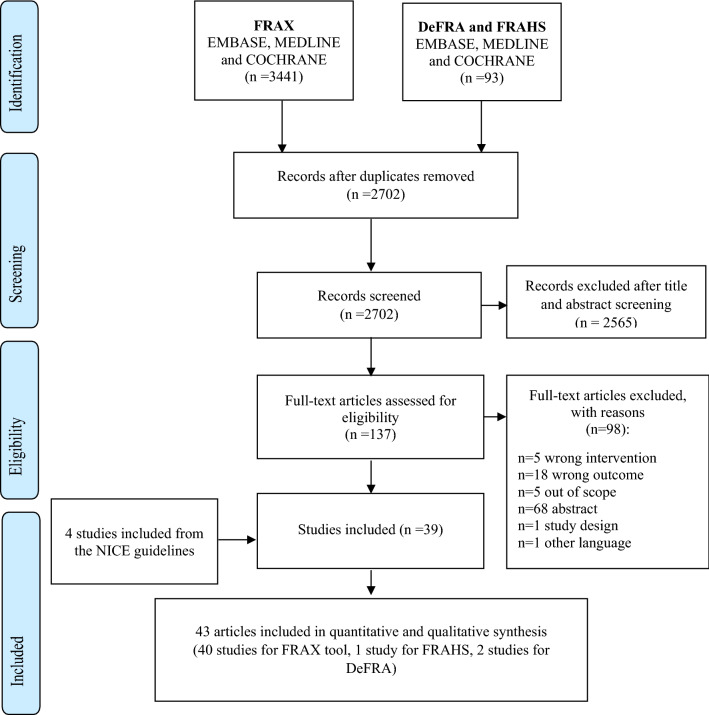
Flow chart

As shown in Fig. 1, a total of 2702 publications were identified; 2565 studies were excluded after title and abstract screening. Among the remaining 137 articles which were assessed for full-text review, 98 were excluded owing to the following reasons: (i) the intervention (n = 5) or outcome (n = 18) was considered to be incorrect, (ii) they were out of scope (n = 5) or only abstract (n = 68), (iii) the study design was not eligible for inclusion (n = 1), and (iv) the studies were not published in the English language (n = 1). Finally, 43 articles were considered for the present analysis; these included 40, 1, and 2 studies pertaining to the FRAX [21–60], FRA-HS [8], and DeFRA [9, 61] tools, respectively.

### Characteristics of included studies

Among the selected articles, 14, 20, and 8 studies had a retrospective [8, 23, 26–28, 33, 35, 36, 43, 53, 59–61], prospective [22, 25, 30–32, 38–42, 47–52, 55–58], and cross-sectional [9, 24, 29, 34, 37, 44–46] designs, respectively; 1 article described a randomized clinical trial [21]. Among the publications, 14 [23–25, 33, 34, 36, 40, 44, 46, 49, 54, 55, 57, 60] were from Asia (Israel, China, Japan, India, Palestine, and Thailand), 21 [8, 9, 22, 26, 28, 29, 31, 35, 37–39, 41–43, 45, 48, 50, 52, 56, 59, 61] were from European countries (Italy, Spain, Poland, France, Denmark, Norway, Portugal, United Kingdom and the Netherlands), 5 [27, 30, 47, 51, 58] were from America, and 3 [21, 32, 53] were from Oceania (Australia, New Zealand). Eleven studies [8, 25, 26, 33, 38–40, 48, 52, 56, 57] considered subjects aged more than 40 years, 2 studies [34, 41] had participants aged over 45 years, 18 publications [9, 23, 24, 27–29, 35, 36, 43, 44, 46, 47, 49–51, 59–61] had individuals aged over 50 years, 4 studies [21, 22, 37, 45] had participants aged over 55 years, 4 publications [32, 42, 53, 58] had subjects aged over 60 years, and 4 studies [30, 31, 54, 55] had participants aged over 65 years. The general characteristics have been presented in Supplemental Material B.

The majority of studies considered subjects with fractures in less than (i) 5% [8, 9, 27, 38, 48, 49, 61], (ii) 10% [23, 24, 36, 40, 41, 43, 46, 47, 52], (iii) 20% [25, 26, 35, 39, 42, 50, 51, 53–55, 60], (iv) 30% [31, 44, 45, 57, 58], and (v) 40% [21, 22, 28–30, 37] cases. One study included participants with previous fractures [59], while four publications did not select subjects with a history of fracture [32–34, 56].

### Risk of bias assessment and certainty of the evidence

Unclear risk of bias was generally present across the studies (Supplemental Table S2). In the entire population, the FRAX tool demonstrated high certainty of evidence: (i) with or without BMD for MOF (at 30% threshold), (ii) MOF (at 20% or 30% cut-off), and (iii) hip fractures (at 3% cut-off, only for Sp) (Supplemental Table S3). A moderate certainty of evidence (Supplemental Table S3) was detected for MOF: (i) without BMD (cut-off at 5% or 20%), (ii) with BMD (at 3% threshold, only for Sn), and (iii) hip fracture with BMD (cut-off at 5%). The remaining Sn and Sp values had low or very low certainty of evidence.

### Sensitivity (Sn) and specificity (Sp)

Sn and Sp evaluation was only performed for the FRAX tool. The results showed a reduction of Sn and an increase of Sp with cut-off augmentation (Table 1, Supplemental Material C).

**Table 1:** Sensitivity (Sn) and specificity (Sp) for major osteoporotic (a) and hip (b) fractures by considering the FRAX tool (with or without BMD) and different cut-off (3%, 5%, 10%, 20%, 30%)

(a) Major osteoporotic fractures										
Cut-off	3%		5%		10%		20%		30%	
FRAX	With BMD (95% CI)	without BMD (95% CI)	With BMD (95% CI)	Without BMD (95% CI)	With BMD (95% CI)	Without BMD (95% CI)	With BMD (95% CI)	Without BMD (95% CI)	With BMD (95% CI)	Without BMD (95% CI)
Women										
Sn	67 [30–93]	57–85 [49–90]	66 [57–73]	34 [27–42]	42–97 [28–98]	46–100 [31–100]	8–41 [2–44]	8 [2–20]	–	4 [0–14]
Sp	75 [63–84]	34–79 [23–82]	71 [67–74]	89 [86–91]	15–84 [14–88]	0–77 [0–81]	81–97 [80–98]	95 [93–97]	99 [97–100]	99 [98–100]
Total										
Sn	–	52–85 [42–90]	–	34–35 [26–44]	42–97 [28–98]	24–100 [16–100]	8–41 [2–44]	8–29 [2–31]	0–9 [0–11]	4–10 [0–14]
Sp		34–79 [23–82]		81–89 [71–91]	15–84 [14–88]	0–93 [0–97]	81–97 [80–98]	88–95 [87–97]	98–99 [97–100]	97–99 [97–100]

(b) Hip fractures					
cut-off	3%		5%		10%
FRAX	With BMD (95% CI)	Without BMD (95% CI)	With BMD (95% CI)	Without BMD (95% CI)	With BMD (95% CI)
Women					
Sn	43–62 [28–64]	8–77 [0–82]	29–76 [19–80]	42–78 [41–82]	33 [28–39]
Sp	72–87 [69–89]	39–100 [36–100]	63–91 [61–94]	50–92 [49–92]	86 [85–87]
Total					
Sn	43–77 [28–81]	8–78 [0–82]	29–76 [19–80]	22–78 [14–82]	–
Sp	72–87 [69–89]	39–100 [36–100]	63–91 [61–94]	50–97 [49–99]	

We reported the minimum and the maximum Sn/Se value, the lower and the upper limit of the 95% confidence interval (CI)

#### Major osteoporotic fractures

In women, the Sn and Sp for FRAX without BMD (and 3% threshold) ranged between 57 and 85% and 34% and 79%, respectively (2 studies [29, 43]). The Sn and Sp for 30% threshold were approximately 4% (95% CI 0–14%) and 99% (95% CI 98–100%), respectively (1 study [42]).

The discriminatory values for FRAX without BMD were lower compared to the predictive values for FRAX with BMD. For FRAX with BMD (and 3% threshold), the estimated Sn and Sp were 67% (95% CI 30–93%) and 75% (95% CI 63–84%), respectively (1 study [23]); the Sp for the 30% threshold was 99% (95% CI 97–100%) (1 study [42]). As showed in Table 1a, the same trend was confirmed in the entire population.

#### Hip fractures

Three studies [21, 33, 56] evaluated the diagnostic accuracy in women (Table 1b); for FRAX without BMD (and 3% threshold), they detected a Sn and Sp ranging from 8 to 77% and 39 to 100%, respectively. For the 5% cut-off value, the Sn and Sp ranged from 42 to 78% and 50 to 92%, respectively (3 studies [21, 30, 56]).

For FRAX with BMD and 3% threshold, the Sn varied from 43 to 62% while the Sp was estimated to be 78–87% (4 studies [21, 23, 27, 42]). For the 10% cut-off value, the Sn was 33% (95% CI 28–39%; 1 study) and the Sp was 86% (95% CI 85–87%; 1 study [27]). As shown in Table 1b, these trends for Sn and Sp (FRAX with or without BMD) were confirmed in the entire population.

### Area under the curve

The meta-analytic summary of the AUCs for the risk assessment tools is shown in Supplemental Material C and Table 2. The diagnostic accuracy of the FRAX (with and without BMD) and FRA-HS tools (without BMD) was evaluated in women, men, and the entire population. The AUC for DeFRA (with BMD) in cases of MOF was evaluated and compared to that of the FRAX instrument in women as well as in diabetic patients.

**Table 2:** Area under the curve (AUC) for major osteoporotic (a) and hip (b) fractures by considering the FRAX, FRA-HS, DeFRA tools (with or without BMD)

(a) Popolation	FRAX (95% CI)		FRA-HS (95% CI)	DeFRA (95% CI)
Women	MOF with BMD	59–88 [54–88]		74 [69–80]
	MOF without BMD	50–78 [57–80]	58 [54–62]	
	HIP with BMD	70–93 [61–100]		
	HIP without BMD	60–86 [56–100]	74 [67–81]	
Men	MOF with BMD	57–85 [41–88]		
	MOF without BMD	55–81 [55–85]	48 [42–54]	
	HIP with BMD	75–90 [72–93]		
	HIP without BMD	57–93 [57–95]	54 [39–69]	
Total	MOF with BMD	57–88 [41–88]		
	MOF without BMD	55–81 [55–85]	65 [61–69]	
	HIP with BMD	70–93 [61–100]		
	HIP without BMD	57–93 [56–100]	73 [66–80]	

(b) Popolation	FRAX		DeFRA
Diabetics	MOF with BMD	73 [60–87]	89 [78–100]

We reported the minimum and the maximum AUC value, the lower and the upper limit of the 95% confidence interval (CI)

In women (Table 2a), the summary AUC of the FRAX (MOF without BMD) indicated a better diagnostic performance (50–78%; 19 studies [21, 22, 25, 29–32, 34, 36, 38–43, 48, 52, 55, 58]) compared with the FRA-HS tool (58%; 1 study [8]); this was reflected in men (55–81% in 5 studies [44, 46, 48, 52, 55] vs. 48% in 1 study [8]) and in the entire population (55–81% in 24 studies [21, 22, 25, 29–32, 34, 36, 38–44, 46–49, 51, 52, 55, 58] vs. 65% in 1 study [8]).

Thus, the summary AUC of the FRAX (hip without BMD) was higher compared to that of the FRA-HS tool in men (57–93% in 6 studies [46, 48, 50, 52, 55, 56] vs. 54% in 1 study [8]); however, no differences were observed in women (60–86% in 17 studies [21, 24, 27, 29–31, 33, 34, 36, 40, 43, 48, 50, 52, 55, 56, 58] vs. 74% in 1 study [8]) and in the entire population (57–93% in 21 studies [21, 24, 27, 29–31, 33, 34, 36, 40, 43, 46–52, 55, 56, 58] vs. 73% in 1 study [8]).

In women, the predictive value of FRAX (MOF with BMD) was similar to that of DeFRA (59–88% in 23 studies [9, 21–23, 25, 26, 28–30, 32, 34, 35, 37, 39–42, 52–55, 57, 59] vs. 74% in 1 study [9]).

In individuals with diabetes, the Italian DeFRA demonstrated a major but non-significant discriminatory value (AUC 89%, 95% CI 78–100%; 1 study [61]) for MOF with BMD with respect to the FRAX tool (AUC: 73%, 95% CI 60–87%; 1 study [61]) (Table 2b).

Inconsistencies, classified as not serious, serious, and very serious, have been presented in Supplementary Table S3.

## Discussion

This systematic review evaluated one clinical question of the Italian Guidelines [11], and a multidisciplinary panel of experts formulated recommendations through a structured, transparent, and evidence-based process. This systematic review and meta-analysis was particularly conducted to evaluate the accuracy of three fracture risk assessment tools (DeFRA, FRAX, and FRA-HS). A total of 43 studies that assessed the performance of tools in identifying at-risk patients were included. Overall, FRAX and DeFRA appeared to perform better than FRA-HS in terms of discriminatory power. All three tools generally performed better for hip fractures than for MOF. As expected, the AUC was higher in women compared to men, mostly with the addition of BMD in the algorithm.

The results of this meta-analysis allowed determination of a recommendation, which suggests the use of risk assessment tools for predicting fractures in patients with or at risk of fragility fractures (moderate quality of evidence).

Other meta-analyses have been conducted on this topic. In 2019, Beaudoin and colleagues published a systematic review and meta-analysis that assessed 14 tools including the FRAX and FRA-HS. The authors analyzed 53 validation studies and found results similar to those of the present meta-analysis. For instance, Beaudoin et al. showed that the tools performed better in predicting hip fractures than fractures at other sites. They also found that the Q-Fracture and Garvan risk tools slightly outperformed the FRAX in predicting hip fractures; this concurs with the findings of an older meta-analysis by Marques and colleagues [62]. In the present meta-analysis, we also found that the DeFRA had slightly higher discriminatory power compared to the FRAX. Indeed, the Garvan, Q-Fracture, and DeFRA tools resolve certain critical issues of the FRAX. Although the FRAX tool represents a crucial milestone in the management of osteoporosis, the algorithm has significant limitations; this may undermine its predictive value. For example, the FRAX does not consider lumbar spine BMD data, which are considered by the DeFRA and Garvan tools. In addition, clinical risk factors (e.g., prior fractures, glucocorticoids, and smoking habits, among others) are scaled down to dichotomous variables in FRAX. However, small differences in prediction ability between FRAX and other more complex algorithms may only have minimal relevance.

### Limitations and strengths

The findings of this study should be interpreted considering its strength and limitations. First, the task force decided to include only three fracture risk assessment tools in the Italian Guideline on the management of Fragility Fracture, because these instruments have been translated into the Italian language. Second, there are certain concerns as to whether findings from selected studies can be combined to draw one conclusion; this is because all the aforementioned results had high levels of heterogeneity depending on the baseline characteristics of the validation cohorts and the quality of the included studies (fracture diagnosis, and length of follow-up, among others). Third, an unclear risk of bias was detected across the included studies. Thus, the certainty of evidence for the assessed outcomes was judged to be “very low” or “moderate” owing to very serious inconsistencies and serious imprecision of the estimates. Fourth, most of the studies included in the meta-analysis were conducted outside Italy and the results might not be directly applicable to the Italian population. However, the vast majority of the population of the meta-analysis was of European ancestry possibly reducing such bias.

Despite these limitations, this study had certain strengths. In view of the discriminatory power of the risk assessment tools, the exhaustive search strategy provided a reliable overview of the studies. In addition, the internal validity of the included studies was assessed using the QUADAS-2 checklist for diagnostic accuracy studies.

## Conclusion

The present meta-analysis evaluated the diagnostic accuracy of three (FRAX, FRA-HS, and DeFRA) fracture risk prediction tools. The task force formulated recommendations on the use of any of these algorithms but did not identify a better performing tool. Although, our systematic review identified some outcomes (Sn and Sp) that were affected by “very low” to “moderate” quality evidence.

### Supplementary Information

Below is the link to the electronic supplementary material.

Supplementary file1 (DOCX 949 KB)

Supplementary file2 (PDF 919 KB)

Supplementary file3 (DOCX 18 KB)

Supplementary file4 (DOCX 47 KB)

## Declarations

### Data sharing

No additional data is available.

### Transparency declaration

The lead author (the manuscript’s guarantor) affirms that the manuscript is an honest, accurate, and transparent account of the study being reported; that no important aspects of the study have been omitted; and that any discrepancies from the study as planned (and, if relevant, registered) have been explained.
