# Supplementary material for: A systematic review on the performance of fracture risk assessment tools: FRAX, DeFRA, FRA-HS
Source: J Endocrinol Invest. 2023 Apr 9;46(11):2287–97. doi: 10.1007/s40618-023-02082-8 (PMC10558377; doi:10.1007/s40618-023-02082-8)
Supplement: Supplementary file 1 — Supplementary file1 (DOCX 949 KB) [file 40618_2023_2082_MOESM1_ESM.docx]

**Supplemental Material**

[A) Search Strategy 1](#_Toc119503537)

[B) Characteristics of included studies 6](#_Toc119503538)

[C) Diagnostic Accuracy 62](#_Toc119503539)

[Sensitivity and Specificity, FRAX 62](#_Toc119503540)

[WOMEN 62](#_Toc119503541)

[POPULATION 63](#_Toc119503542)

[WOMEN and POPULATION 65](#_Toc119503543)

[Area under the curve, FRAX 67](#_Toc119503544)

[WOMEN 67](#_Toc119503545)

[MEN 69](#_Toc119503546)

[POPULATION 71](#_Toc119503547)

[WOMEN, MEN AND POPULATION 73](#_Toc119503548)

[Area under the curve, FRA-HS 75](#_Toc119503549)

[WOMEN, MEN OR BOTH 75](#_Toc119503550)

[Area under the curve, DeFRA and FRAX 75](#_Toc119503551)

[WOMEN 75](#_Toc119503552)

[DIABETIC POPULATION 76](#_Toc119503553)

[D) Complete list of experts involved 77](#_Toc119503554)

# Search Strategy

Review question 2: Which risk assessment tools are the most accurate in predicting the risk of fragility fracture in adults, including those without known osteoporosis or previous fragility fracture?


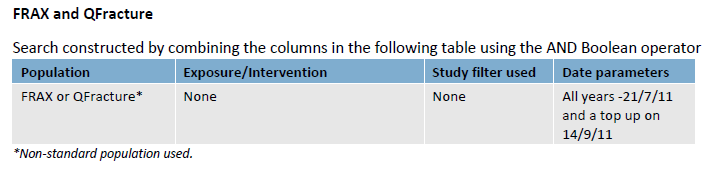


Up to 7 December 2020

**MEDLINE SEARCH**: FRAX TOOL

#1: FRAX[tiab] or FRAXTM[tiab]

#2: risk*[tiab] and assess*[tiab] and tool*[tiab]

#3: fracture*[tiab]

#4: #2 AND #3:

#5: “fracture risk assessment tool”[tiab]

#6: 1 or 4 or 5

#7: Letter/

#8: Editorial/

#9: News/

#10: exp Historical article/

#11: Anecdotes as topic/

#12: Comment/

#13: Case report/

#14: Letter[ti] or comment*[ti] or abstracts[ti]

#15: or/7-14

#16: #6 not #15 Filters: Humans, from 2011/9/14

**EMBASE search**

#1: frax:ti,ab OR fraxtm:ti,ab

#2: risk*:ti,ab AND assess*:ti,ab AND tool*:ti,ab

#3: fracture*:ti,ab

#4: #2 and #3

#5: “fracture risk assessment tool”:ti,ab

#6: 1 or 4 or 5

#7: letter.pt. or Letter

#8: note.pt

#9: editorial.pt

#10: (Case report) or (Case study)

#11: letter:ti or comment*:ti

#12: #7 OR #8 OR #9 OR #10 OR #11

#13: “Randomized controlled trial”:ti,ab or random*:ti,ab

#14: #12 not #13

#15: Animal not Human

#16: Nonhuman

#17: exp Animal experiment

#18: exp Experimental animal

#19: Animal model

#20: exp Rodent

#21: Rat:ti or rats:ti or mouse:ti or mice:ti

#22 #14 or #15 or#16 or #17 or #18 or #19 or #20 or #21

#23: #6 not #22

#24: 2,279 articles

#23 AND (2011:py OR 2012:py OR 2013:py OR 2014:py OR 2015:py OR 2016:py OR 2017:py OR 2018:py OR 2019:py OR 2020:py OR 2021:py) AND [embase]/lim NOT ([embase]/lim AND [medline]/lim)

**COCHRANE SEARCH**

#1: (FRAX or FRAXTM):ti,ab,kw

#2: (fracture* risk assess* tool*):ti,ab,kw

#3: (risk* and assess* and tool*):ti,ab

#4: fracture*:ti,ab

#5: (#3 AND #4)

#6. 318 articles

(#1 OR #2 OR #5) with Cochrane Library

Up to 8 December 2020

**MEDLINE SEARCH**: DEFRA, FRA-HS TOOL

#1: DEFRA[tiab] or FRA-HS[tiab] or FRAHS[tiab]

#2: Letter/

#3: Editorial/

#4: News/

#5: exp Historical article/

#6: Anecdotes as topic/

#7: Comment/

#8: Case report/

#9: Letter[ti] or comment*[ti] or abstracts[ti]

#10: or/2-9

#11: 46 articles

#1 not #10 Filters: Humans

**EMBASE search**

#1: defra:ti,ab OR frahs:ti,ab or fra-hs:ti,ab

#2: letter.pt. or Letter

#3: note.pt

#4: editorial.pt

#5: (Case report) or (Case study)

#6: letter:ti or comment*:ti

#7: #2 OR #3 OR #4 OR #5 OR #6

#8: “Randomized controlled trial”:ti,ab or random*:ti,ab

#9: #7 not #8

#10: Animal not Human

#11: Nonhuman

#12: exp Animal experiment

#13: exp Experimental animal

#14: Animal model

#15: exp Rodent

#16: Rat:ti or rats:ti or mouse:ti or mice:ti

#17 #9 or#10 or #11 or#12 or #13 or #14 or #15 or #16

#18: #1 not #17

#19: 45 articles#18 AND [embase]/lim NOT ([embase]/lim AND [medline]/lim)

**COCHRANE SEARCH**

#1: 2 articles

(defra or frahs or fra-hs):ti,ab,kw

# Characteristics of included studies

| **Study** | **External validation and comparison of three prediction tools for risk of osteoporotic fractures using data from population based electronic health records: retrospective cohort study**  **Dagan 2017** |
| --- | --- |
| Study type | Retrospective cohort study. |
| Number of studies/ number of participants | N= 1054815 |
| Countries and Settings | This study used electronic health record data from Clalit Health Services, the largest of four national health funds in Israel. |
| Funding | Not reported |
| Duration of study | From 2010 to 2014. |
| Age, gender, ethnicity, baseline fracture | Study population: aged 50-59 38%; aged 60-69 28.4%; aged 70-79 21.1%; aged 80-89 12.5%.  Sex: Men 45.4%; women 54.6%.  Ethnicity: black Africans 1.2%; white 98.8%.  Baseline fracture: 156340 (14.8%) |
| Patient characteristics | The comparative analysis was performed among members of Clalit Health Services aged 50 to 90 years as of the index date, who had at least three years of continuous membership before the index date and through the follow-up period or until death. |
| Intervention | We computed the five year risk according to QFracture (2012 version) and Garvan based on their full tool equations. Since the current FRAX equations are not published by the authors, we used the FRAX 10 year probability charts calibrated for Israel, stratified by sex, age, body mass index, and number of clinical risk factors, as supplied by the official FRAX site. |
| Outcomes | Outcome variables included both hip fracture and major osteoporotic fractures, which were defined as fractures of the hip, vertebrae, distal radius, or proximal humerus. These variables were defined based on the records for clinical diagnoses. |

| **Study** | **Fracture prediction and calibration of a Canadian FRAX® tool: a population-based report from CaMos**  **Fraser 2011** |
| --- | --- |
| Study type | Prospective population based cohort study |
| Number of studies/ number of participants | N= 6697 |
| Countries and Settings | Canadian Multi-centre Osteoporosis Study (CaMos). |
| Funding | Not reported |
| Duration of study | Not reported |
| Age, gender, ethnicity, baseline fracture | Women 4778, Age 65.8 ± 8.8  Men 1919, Age 65.3 ± 9.1  634 (9.5%) patients have baseline fracture |
| Patient characteristics | We included all CaMos participants, with follow-up data, aged ≥50 years at study entry. Briefly, eligible participants were at least 25 years old at the start of the study, lived within a 50-km radius of one of nine Canadian cities and were able to converse in English, French or Chinese. |
| Intervention | The WHO Coordinating Centre used the Canadian FRAX tool calibrated using national hip fracture and mortality data along with the FRAX predictor variables from CaMos to calculate 10-year fracture probability. |
| Outcomes | Self-reported incident clinical fractures were identified by yearly postal questionnaire or at the scheduled interval for in-person reassessment (third, fifth and tenth year after study entry). |

| **Study** | **Fracture Risk Prediction Using Phalangeal Bone Mineral Density or FRAX? A Danish Cohort Study on Men and Women**  **Friis-Holmberg 2014** |
| --- | --- |
| Study type | Prospective cohort study |
| Number of studies/ number of participants | N= 12758 |
| Countries and Settings | Danish Health Examination Survey |
| Funding | Not reported |
| Duration of study | 2007-2012 |
| Age, gender, ethnicity, baseline fracture | Men 5206, mean age 58.3 ± 10.6, 443 (5.9%) have baseline fracture  Women 7552, mean age 56.8 ± 10.2, 132 (2.5%) have baseline fracture |
| Patient characteristics | We used data on a cohort of women and men aged 18-95 yr who participated in the Danish Health Examination Survey 2007-2008. The present study includes data from participants aged 40-90 yr (i.e., the applied age range in FRAX), who had a BMD scan. Participants were excluded if height or weight was missing. |
| Intervention | As the algorithm for FRAX is unpublished, the 10-yr risk of fracture was calculated by individual risk scoring of the Danish version of FRAX using a programed call of the FRAX Web site. |
| Outcomes | Incident fractures were defined as fractures occurring between the date of BMD measurement in 2007-2008 and end of follow-up (10th of August 2012) and calculated as the number of persons with a fracture during the follow-up period. Prevalent fractures were defined as fractures occurring before the date of BMD measurement. Hip fractures were validated and excluded if no corresponding surgical code of primary hip arthroplasty or osteosynthesis |
| **Study** | **Validation of FRAX and the impact of self-reported falls among elderly in a general population: the HUNT study, Norway**  **Hoff 2017** |
| Study type | Observational study |
| Number of studies/ number of participants | N= 29017 |
| Countries and Settings | third survey of the Nord-Trøndelag Health Study (HUNT3), fracture registry in Nord-Trøndelag, and Norwegian Prescription Database (NorPD) |
| Funding | Not reported |
| Duration of study | 1995 – 2016 |
| Age, gender, ethnicity, baseline fracture | Men n.13585, mean age 64.0 (SD 9.3), 1365 (10%) have baseline fracture  Women n.15432, mean age 64.4 (SD 9.7), 2779 (18%) have baseline fracture |
| Patient characteristics | Aged 50-90 years. |
| Intervention | FRAX estimates 10-year osteoporotic and hip fracture probability. The Norwegian FRAX tool was recalibrated based on Norwegian data on incidence of hip fracture and mortality, and the FRAX scores were calculated on the basis of FRAX Desktop |
| Outcomes | A fracture was defined when (1) the ICD code was accompanied by a medical record confirmation of hip fracture or (2) a fracture was diagnosed by X-ray. Fractures due to metastatic disease were excluded. |

| **Study** | **Assessing Risk of Osteoporotic Fractures in Primary Care: Development and Validation of the FRA-HS Algorithm**  **Lapi 2017** |
| --- | --- |
| Study type | Cohort study |
| Number of studies/ number of participants | N= 407771 |
| Countries and Settings | the Health Search—IMS Health Longitudinal Patients Database (HSD), an Italian general practice database that includes patients’ records of a group of over 1000 GPs homogenously distributed across Italy. |
| Funding | Not reported |
| Duration of study | 1999-2012 |
| Age, gender, ethnicity, baseline fracture | Mean age 60.08 (SD 12.80)  Males 183308 (females 224359)  Baseline fracture: 114 (1.6%) |
| Patient characteristics | We formed a cohort of patients aged 40 years and over during the period between January 1, 1999 and December 31, 2002. To be considered eligible, patients were required to have at least 1-year medical history in the database. |
| Intervention | We therefore developed and validated the FRActure Health Search (FRA-HS) score, a FRAX®-based model, for the assessment of risk of osteoporotic fractures in primary care in Italy. |
| Outcomes | We identifed all diagnoses coded via ICD9CM which were consistent with osteoporotic fractures occurred during follow-up. Namely, hip/femur (ICD9CM code: 820*, 821.0, 821.2), vertebral (ICD9CM code: 805*), humeral (ICD9CM code: 812*), wrist/forearm (ICD9CM code: 813*) fractures were defined as the study outcome. |
| **Study** | **Independent Clinical Validation of a Canadian FRAX Tool: Fracture Prediction and Model Calibration**  **Leslie 2010** |
| Study type | Cohort study |
| Number of studies/ number of participants | N= 39603 |
| Countries and Settings | the Canadian Institute for Health Information (CIHI) collects and analyzes information on health and health care in Canada and makes this publicly available. The Hospital Morbidity Database (HMDB), a database housed at CIHI, includes administrative, clinical, and demographic information on hospital inpatient events and provides national discharge statistics from Canadian health care facilities by diagnoses and procedures. |
| Funding | Not reported |
| Duration of study | 1990 – 2008 |
| Age, gender, ethnicity, baseline fracture | Men (n.2873) were slightly older than women (n.36730) (mean age 68.2 (10.1) versus 65.7 (9.8), p < .001), and present more baseline fracture (15% vs 13.6%) |
| Patient characteristics | The population for this historical cohort study consisted of all women and men in the Province of Manitoba, Canada, aged 50 years or older at the time of baseline femoral neck dual energy X-ray absorptiometry (DXA) between January 1990 and March 2007. Subjects were required to have medical coverage from Manitoba Health during the observation period ending March 2008 without other exclusions. |
| Intervention | the FRAX estimates using BMD and clinical risk factors |
| Outcomes | Longitudinal health service records were assessed for the presence of hip, clinical vertebral, forearm, and humerus fracture codes |

| **Study** | **Prognosis of fracture: evaluation of predictive accuracy of the FRAXTM algorithm and Garvan nomogram**  **Sandhu 2010** |
| --- | --- |
| Study type | Retrospective validation study |
| Number of studies/ number of participants | N= 200 |
| Countries and Settings | Fracture and Bone and Calcium clinics at St. Vincent’s Hospital, Sydney |
| Funding | Not reported |
| Duration of study | Not reported |
| Age, gender, ethnicity, baseline fracture | Women n.144, of which 69 with fractures, mean age 73 (SD 8), and 75 without fractures, mean age 68 (SD 8), 33 (22.9%) with baseline fracture  Men n.56, of which 31 with fractures, mean age 75 (SD 10), and 25 without fractures, mean age 68 (SD 8), 5 (8.9%) baseline fracture |
| Patient characteristics | Patients were included if they were of Caucasian origin and aged between 60 and 90 years old. |
| Intervention | Using the FRAXTM and FractureRiskCalculator.com websites, we calculated the 10-year risk of fracture for each individual. |
| Outcomes | Fracture cases were included if they had a major osteoporotic fracture as defined in FRAX^TM^ |

| **Study** | **The added value of trabecular bone score to FRAX® to predict major osteoporotic fractures for clinical use in Chinese older people: the Mr. OS and Ms. OS cohort study in Hong Kong**  **Su 2017** |
| --- | --- |
| Study type | Case-control study |
| Number of studies/ number of participants | N= 3873 |
| Countries and Settings | Mr. OS and Ms. OS Hong Kong study. |
| Funding | Not reported |
| Duration of study | 2001 – 2009 |
| Age, gender, ethnicity, baseline fracture | Men 1923, mean age 72.29 (SD 4.87), 262 (13.6%) with baseline fracture  Women 1950, mean age 72.52 (SD 5.26), 403 (20.7%) with baseline fracture |
| Patient characteristics | At baseline, 2000 Chinese men and 2000 Chinese women 65 years old or above were recruited from local communities via advertisements distributed within housing estates and community centers for older people from August 2001 to March 2003. Stratified sampling was utilized to generate a sample with roughly one-third between the ages of 65 and 69, one-third between 70 and 74, and the final third 75 or older. To be eligible, subjects needed to dwell in the community, to be able to walk without assistance. |
| Intervention | The baseline assessment consisted of an interview which used a standardized, structured questionnaire. Information on clinical risk factors in FRAX® was collected. |
| Outcomes | Fracture cases were included if they had a major osteoporotic fracture as defined in FRAX^TM^ |

| **Study** | **Sarcopenia Combined With FRAX Probabilities Improves Fracture Risk Prediction in Older Chinese Men**  **Yu 2014** |
| --- | --- |
| Study type | Prospective cohort study |
| Number of studies/ number of participants | N=4000 |
| Countries and Settings | School of Public Health of the Chinese University of Hong Kong |
| Funding | Not reported |
| Duration of study | 2001 – 2003 |
| Age, gender, ethnicity, baseline fracture | Men n.2000, of which 1774 without fractures, mean age 72.19 (SD 4.92), and 226 with fractures, mean age 74.00 (SD 5.36), 274 (13.7%) with baseline fracture  Women n.2000, of which 1661 without fractures, mean age 72.29 (SD 5.30), and 339 with fractures, mean age 74.01 (SD 5.41), 416 (20.8%) with baseline fracture |
| Patient characteristics | Four thousand community-dwelling Chinese men and women at least 65 years old were invited. Those who were unable to walk independently, had a history of bilateral hip replacements, were not competent to give informed consent, or had medical conditions judged by the study physicians on the likelihood that they would not survive the duration of the primary study were excluded. |
| Intervention | The WHO 10-year absolute risks of both major osteoporotic fracture and hip fracture (FRAX scores) were calculated by the WHO Collaborating Center for Metabolic Bone Disease, using the FRAX algorithm (Hong Kong version) |
| Outcomes | Incident fractures were documented based on history and X-rays during an average of 10.2 years of follow-up (2001-2013). Fracture occurrence was determined by 4 monthly telephone calls and visits to the research center at 2 yearly intervals. The diagnosis of fracture was verified by carrying out a search of the Hospital Authority electronic database, which covers over 95% of all hospital admissions in Hong Kong. This is a computer system containing all hospital discharge summaries and outpatient consultation episodes, including diagnosis and coding. These were further validated by a review of individual medical records. |

| **Study** | **Do we need bone mineral density to estimate osteoporotic fracture risk? A 10-year prospective multicentre validation study**  **Marques 2017** |
| --- | --- |
| Study type | Cohort study |
| Number of studies/ number of participants | N= 2626 |
| Countries and Settings | Data of three different Portuguese cohorts, SAOL, IPR and EPIPorto (from centre, south and north of the country, respectively), were combined. |
| Funding | This study was supported by unrestricted grants from the Direção Geral da Saúde and Amgen, which had no role in the design of the study, the writing or review of the paper. |
| Duration of study | Not declared |
| Age, gender, ethnicity, baseline fracture | Men 683, women 1943  Mean age 58.2 (SD 10.2)  Baseline fracture: 512 (19.5%) |
| Patient characteristics | Only persons aged >40 years and with a complete set of data on FRAX® clinical risk factors were included. There were no other exclusion criteria. |
| Intervention | The 10-year fracture risk estimates for hip and MOP fractures (with and without adding the variable femoral neck BMD) |
| Outcomes | The first new fracture during follow-up and the date on which it occurred were self-reported at the follow-up visit in all cohorts. |
| **Study** | **Predicting risk of osteoporotic fracture in men and women in England and Wales: prospective derivation and validation of QFractureScores**  **Hippisley-Cox 2009** |
| Study type | Prospective cohort study |
| Number of studies/ number of participants | N= 1183663 women and 1174232 men |
| Countries and Settings | General practices in England and Wales. |
| Funding | This study was funded by David Stables (medical director of EMIS) as part of a larger study examining risks and benefits of HRT. |
| Duration of study | 1993 – 2008 |
| Age, gender, ethnicity | 1 183 663 women and 1 174 232 men aged 30-85 in the derivation cohort. |
| Patient characteristics | Only persons aged >40 years and with a complete set of data on FRAX® clinical risk factors were included. There were no other exclusion criteria. |
| Intervention | We took the regression coefficient for each variable from the final model using multiply imputed data and used these as weights for the QFractureScores. We restricted our comparative analysis to the hip fracture outcome as this is directly comparable between both scores, whereas the FRAX fracture outcome also includes humerus fractures. We used the UK version of the score from the FRAX website to calculate the 10 year predicted risk of hip fracture |
| Outcomes | First (incident) diagnosis of osteoporotic fracture (vertebral, distal radius, or hip) and incident hip fracture recorded in general practice records. |

| **Study** | **Fracture risk prediction in outpatients from Krakox region using FRAX tool versus fracture risk in 11-year follow-up**  **Czerwiński 2013** |
| --- | --- |
| Study type | Retrospective cohort study |
| Number of studies/ number of participants | N= 1024 |
| Countries and Settings | Cracow Medical Centre |
| Funding | Not reported |
| Duration of study | Between 1997 and 2008 |
| Age, gender, ethnicity, baseline fracture | Age [mean (range)]: 50 years and above  Gender (n. F): Intervention group:100%; Placebo group: 100%  Baseline fracture: 324 (32%) |
| Patient characteristics | The study involved Caucasian women aged ≥ 50 and ≤ 80 years at baseline who were residents of Malopolska region, who gave their oral consent to participate in the study and who, in the opinion of the interviewer, were capable of accurately answering the 15-minute phone questionnaire. Each patient underwent a densitometric examination of the spine and/or proximal femur at the time of the first survey. Patients with any physical or mental disorders which could influence memory and credibility of the acquired data (hearing loss, dementia or any memory impairment, aphasia that impeded communication) as well as patients who did not completely respond to questions in the second questionnaire or refused to continue answering the questions and did not wish to repeat the questionnaire were excluded from the study. |
| Intervention | During a patient’s first visit to the Centre, trained medical staff obtained demographic and anthropometric data and medical history, including co-morbidities, family history and use of medications. A dedicated part of the questionnaire was devoted to fracture risk factors: occurrence of falls in the preceding one and five years, past fractures and diagnosis and treatment of osteoporosis.  After an average of 11 years, a telephone survey was conducted among a randomly selected group of patients using a questionnaire corresponding to the one applied in the first survey. Special attention was paid to the occurrence of fractures  It was used the FRAX tool with BMD |
| Outcomes | - To assess the predictive value of FRAX tool - To assess the prevalence of clinical risk factors and, in particular, of osteoporotic fractures |

| **Study** | **Assessment of Fracture Risk in A Population of Postmenopausal Italian Women: A Comparison of Two Different Tools**  **Bonaccorsi 2015** |
| --- | --- |
| Study type | cross-sectional population-based study |
| Number of studies/ number of participants | N= 989 |
| Countries and Settings | Ferrara, Italy |
| Funding |  |
| Duration of study | Between 2012 and 2013 |
| Age, gender, ethnicity | The mean age of the population (n = 989) was 63.6 years (range 50–89).  Baseline fracture: 24 (2.4%) |
| Patient characteristics | Women of Caucasian origin aged between 50 and 90 years old. Subjects were excluded if they had been on osteoporosis treatment for more than 3 years at the time of evaluation |
| Intervention | The individual 10-year fracture risk was assessed by the FRAX tool for Europe-Italy (available online at the website http://www.shef.ac.uk/FRAX) considering CRFs for osteoporosis collected in the case history and the T-score for femoral neck BMD. DeFRA was also used to evaluate the individual 10-year fracture risk (website https://DeFRA-osteoporosi.it). It was possible to calculate DeFRA-risk only for the female population aged 50 years or more by inputting the lowest T-score between femoral and vertebral BMD. |
| Outcomes | The output is the 10-year probability of a major osteoporotic fracture (clinical vertebral, hip, forearm, or proximal humerus fractures).population. |

| **Study** | **A non-invasive prevention program model for the assessment of osteoporosis in the early postmenopausal period: a pilot study on FRAX and QUS tools advantages**  **Villa 2016** |
| --- | --- |
| Study type | Retrospective cohort study |
| Number of studies/ number of participants | N= 282 |
| Countries and Settings | Italy |
| Funding | Not reported |
| Duration of study | Between 2012 and 2013 |
| Age, gender, ethnicity, baseline fracture | Age [mean (range)]: 54 years and above  Gender (n. F): Intervention group: 100%; Placebo group: 100%  Baseline fracture: 20 (7.1%) |
| Patient characteristics | The study included women who were consecutively enrolled in the clinical center, who were within 10 years after menopause. Women with evidence and diagnosis of severe metabolic bone diseases were excluded, as well as women with history of cancer, severe renal impairment, or abnormal liver function. |
| Intervention | All the patients filled out a questionnaire to determine their FRAX index by expert personnel. Thereafter, they underwent the bone assessments first by QUS of the heel using Achilles In Sight device and then by DXA exam at the femoral neck and lumbar spine level. The personnel performing both FRAX and QUS examinations were blinded to the DXA results and vice versa. FRAX results were then compared to QUS and DXA without adding BMD.  It was used the FRAX tool without BMD |
| Outcomes | - To analyze the performances of FRAX algorithm and quantitative ultrasound (QUS) tool in relationship to the dual-energy X-ray absorptiometry categorization to identify patients at risk of osteoporosis during menopause and to reach new thresholds for recommending the first DXA examination |

| **Study** | **Comparison of different screening tools (FRAX, OST, ORAI, OSIRIS, SCORE and age alone) to identify women with increased risk of fracture. A population-based prospective study**  **Rubin 2013** | |
| --- | --- | --- |
| Study type | Prospective population-based study | |
| Number of studies/ number of participants | N= 3614 | |
| Countries and Settings | Region of Southern Denmark, Danish National Patient Register (NPR), Danish National Civil Registration System (NCR) | |
| Funding | This study was supported by INTERREG 4A (JNR 08/5177), the Region of Southern Denmark (JNR 08/8133 and JNR 11/5761) and Odense University Hospital | |
| Duration of study | Between 2009 and 2012 | |
| Age, gender, ethnicity, baseline fracture | Age [mean (range)]: 64 years and above  Gender (% F): Intervention group: 100%; Placebo group: 100%  Baseline fracture: 156 (4.3%) | |
| Patient characteristics | Women aged 40-90 years, stratified by decades and who returned a questionnaire were included in the analyses, with the exception of those diagnosed with and treated for osteoporosis | |
| Intervention | Fracture risk was calculated using the different screening tools for each woman. The women were followed during 3 years, counting only the first fracture per person  It was used the FRAX tool without BMD | |
| Outcomes | - To compare the power of FRAX without bone mineral density and simpler screening tools in predicting fractures | |
| **Study** | **Evaluation of the FRAX and Garvan Fracture Risk Calculators in Older Women**  **Bolland 2011** |  |
| Study type | Randomized placebo-controlled trial |  |
| Number of studies/ number of participants | N= 1422 |  |
| Countries and Settings | New Zealand |  |
| Funding | This study was funded by grants from the Health Research Council of New Zealand |  |
| Duration of study | Between 2001 and 2007 |  |
| Age, gender, ethnicity, baseline fracture | Age [mean (range)]: 55 years and above  Gender (n. F): Intervention group:100%; Placebo group: 100%  Baseline fracture: 33.5% |  |
| Patient characteristics | The study involved women older than 55 years of age, free from major medical conditions, with normal lumbar spine bone mineral density for their age, who were not taking treatment for osteoporosis in doses > 1000 IU/day, and had serum 25(OH)D levels ≥ 25 nmol/L.  Were excluded women for whom measurements of femoral neck BMD at baseline were missing, and for whom no further data were available after the baseline visit. |  |
| Intervention | All surviving study partecipants were contacted by telephone, and details of any fractures and other medical events since study completion were recorded.  It was used the FRAX-New Zealand tool |  |
| Outcomes | - To assess the performance of the FRAX and Garvan fracture risk calculators |  |

| **Study** | **FRAX: Prediction of Major Osteoporotic Fractures in Women from the General Population: The OPUS Study**  **Briot 2013** |
| --- | --- |
| Study type | Prospective study |
| Number of studies/ number of participants | N= 1748 |
| Countries and Settings | OPUS study |
| Funding | The OPUS cohort was sponsored by Eli Lilly, Sanofi-Aventis, Procter and Gamble Pharmaceuticals, Hoffman-La Roche, Pfizer and Novartis |
| Duration of study | Between 1999 and 2001 |
| Age, gender, ethnicity, baseline fracture | Age [mean (range)]: 55 years and above  Gender (n. F): Intervention group:100%; Placebo group: 100%  Baseline fracture: 742 (42.4%) |
| Patient characteristics | The study involved European women aged above 55 years with information on incident major osteoporotic fractures. Women were excluded if they had disorders precluding ultrasound and bone mineral density measurements, and also general and cognitive inability that precluded completing questionnaire. |
| Intervention | The predictive value of FRAX was analysed in the whole population and in a subgroup of 698 patients who had never been treated before or during the study.  It was used the FRAX with and without BMD |
| Outcomes | - To analyse how well FRAX predicts the risk of major osteoporotic and vertebral fractures |

| **Study** | **A comparison of prediction models for fractures in older women: is more better**  **Ensrud 2009** |
| --- | --- |
| Study type | Prospective cohort study |
| Number of studies/ number of participants | N= 6252 |
| Countries and Settings | United States |
| Funding | The Study of Osteoporotic Fractures (SOF) is supported by National Institutes of Health funding |
| Duration of study | Between 1986 and 1990 |
| Age, gender, ethnicity, baseline fracture | Age [mean (range)]: 65 years and above  Gender (n. F): Intervention group:100%; Placebo group: 100%  Baseline fracture: 2155 (34.5%) |
| Patient characteristics | The study involved women aged at least 65 years old, from population-based listing in four areas of the United States. Black women were originally excluded from SOF because of their low incidence of hip fracture. In addition, women were excluded if they were unable to walk without assistance or had a history of bilateral hip replacement. |
| Intervention | Participants completed a questionnaire and were interviewed at the baseline examination and asked about race/ethnicity, prior history of fracture since the age of fifty years, physician diagnosis of rheumatoid arthritis, parental history of hip fracture, smoking status, alcohol intake, and use of glucocorticoids.  It was used the FRAX tool with BMD |
| Outcomes | - To determine whether prediction with FRAX models is superior to that based on parsimonious models |

| **Study** | **Fracture risk prediction score and absolute risk of fracture**  **Henry 2011** |
| --- | --- |
| Study type | Population-based age-stratified random sample study |
| Number of studies/ number of participants | N= 600 |
| Countries and Settings | Barwon Statistical Division |
| Funding | This study received institutional grants from National Health and Medical Research Council, Victorian Health Promotion Foundation, and Geelong Region Medical Research Foundation |
| Duration of study | Between 1996 and 2006 |
| Age, gender, ethnicity | Age [mean (range)]: 60 years and above  Gender (n. F): Intervention group:100%; Placebo group: 100% |
| Patient characteristics | The study involved white women who were 60 years and older |
| Intervention | It was used FRAX UK, and FRAX US |
| Outcomes | - To report the 5- and 10-year absolute risk of fracture associated with the previously reported fracture risk (FRISK) |

| **Study** | **Ten-year probability of osteoporotic fracture in 2012 Polish women assessed by FRAX and nomogram by Nguyen et al.-Conformity between methods and their clinical utility**  **Pluskiewicz 2010** |
| --- | --- |
| Study type | Cross-sectional study |
| Number of studies/ number of participants | N= 2012 |
| Countries and Settings | Poland |
| Funding | Not reported |
| Duration of study | Between 2008 and 2009 |
| Age, gender, ethnicity, baseline fracture | Age [mean (range)]: 55 years and above  Gender (n. F): Intervention group:100%; Placebo group: 100%  Baseline fracture: 728 (36.2%) |
| Patient characteristics | The study involved postmenopausal women aged 55 years and older |
| Intervention | The studied group was divided into:   - two fracture risk thresholds in case of any fracture (≤20% and > 20%) - two fracture risk thresholds in case of hip fracture (≤3% and >3%)   It was used FRAX tool with BMD |
| Outcomes | - To establish the degree of conformity between 10-year probability of osteoporotic fracture |

| **Study** | **The FRAX tool in French women: how well does it describe the real incidence of fracture in the OFELY cohort**  **Sornay-Rendu 2010** |
| --- | --- |
| Study type | Prospective study |
| Number of studies/ number of participants | N= 867 |
| Countries and Settings | Rhône District |
| Funding | Not reported |
| Duration of study | Between 1992 and 1993 |
| Age, gender, ethnicity, baseline fracture | Age [mean (range)]: 40 years and above  Gender (n. F): Intervention group:100%; Placebo group: 100%  Baseline fracture: 89 (10.3%) |
| Patient characteristics | The study involved French women aged 40 years or more at the inclusion in the study |
| Intervention | Women were randomly selected from the affiliates of a large health insurance company with an annual follow-up. Women completed a questionnaire at the initial screening visit, including all clinical risk factors used in calculation if the predicted fracture probability with the FRAX tool.  It was used the FRAX tool with and without BMD |
| Outcomes | - To compare the predicted fracture probabilities and the observed incidence of fracture in French women during a 10-year follow-up |

| **Study** | **Fracture risk prediction using FRAX: a 10-year follow-up survey of the Japanese population-based osteoporosis (JPOS) cohort study**  **Tamaki 2011** |
| --- | --- |
| Study type | Prospective cohort study |
| Number of studies/ number of participants | N= 815 |
| Countries and Settings | Japan |
| Funding | Financial support for the baseline survey was provided by the Japan Milk Promotion Board and the Japan Dairy Council. The follow-up surveys were supported by Grants-in-aid for Scientific Research from the Japanese Society for the Promotion of Science, a grant in 2000-2002 from the Research Society for Metabolic Bone Diseases, Japan, and a Grant-in-aid to study Milk Nutrition from the Japan Dairy Association |
| Duration of study | Between 1996 and 2001 |
| Age, gender, ethnicity, baseline fracture | Age [mean (range)]: 40 years and above  Gender (n. F): Intervention group:100%; Placebo group: 100%  Baseline fracture: 65 (8%) |
| Patient characteristics | The study included women aged 40 years and older. Sixty-nine women were excluded from the analysis because of the low follow-up rate. Women who did not have femoral neck bone mineral density measurements at the baseline survey and women taking osteoporosis drugs or hormone replacement therapy at the baseline survey were excluded. |
| Intervention | Women were randomly selected from 5-year age groups using resident registrations in seven municipalities throughout Japan. Women from three areas of those municipalities were selected as the cohort. There was mailed a questionnaire on osteoporotic fracture events to women who did not participate in the 10-year follow-up study.  It was used the FRAX tool with and without BMD. |
| Outcomes | - To evaluate the ability of the Japanese version of FRAX to predict the 10-year probability of osteoporotic fractures using follow-up data from the prospective JPOS |

| **Study** | **Fracture risk prediction using BMD and clinical risk factors in early postmenopausal women: sensitivity of the WHO FRAX tool**  **Trémollieres 2010** |
| --- | --- |
| Study type | Prospective cohort study |
| Number of studies/ number of participants | N= 2651 |
| Countries and Settings | Menopause et Os (MENOS) cohort study, Menopause Center of the Toulouse University Hospital |
| Funding | This work was part of the MENOS study and was supported by an institutional grant from Lilly France and Pierre Fabre Santé Laboratories |
| Duration of study | Between 1988 and 1991 |
| Age, gender, ethnicity, baseline fracture | Age [mean (range)]: 45 years and above  Gender (n. F): Intervention group:100%; Placebo group: 100%  Baseline fracture: 145 (6.6%) |
| Patient characteristics | The study involved postmenopausal women 45 years and older. Women were considered postmenopausal if they had not menstruated within the last 12 months before the examination, associated with serum follicle-stimulating hormone levels above 30 IU/L and serum estradiol levels below 20 pg/mL.  Women with past/current osteoporosis treatment for more than 3 months at baseline were excluded from the analyses. |
| Intervention | At baseline, all women answered a computer-assisted standardized questionnaire recorded by the same trained research nurse. At the follow-up visit, anthropometric measurements were taken, and all women answered the MENOS epidemiologic standardized questionnaire |
| Outcomes | - To identify significant and independent clinical risk factors (CRFs) for major osteoporotic fracture among peri- and early postmenopausal women - To assess the discriminatory capacity of FRAX and bone mineral density for the identification of women at high risk of fracture - To assess whether adding risk factors to either FRAX or BMD would improve discriminatory capacity |

| **Study** | **The utility of absolute risk prediction using FRAX and Garvan fracture risk calculator in daily practice**  **van Geel 2014** |
| --- | --- |
| Study type | Prospective cohort study |
| Number of studies/ number of participants | N= 506 |
| Countries and Settings | The Netherlands |
| Funding | Not reported |
| Duration of study | Between 1992 and 1994 |
| Age, gender, ethnicity, baseline fracture | Age [mean (range)]: 60 years and above  Gender (n. F): Intervention group:100%; Placebo group: 100%  Baseline fracture: 134 (26.5%) |
| Patient characteristics | The study involved women 60 years and over who were registered in one of 10 general practice centres of whom all risk factors needed to complete both risk prediction tools were available |
| Intervention | A questionnaire enquired about possible risk factors related to fractures. Five years later, a questionnaire was completed by all participating women regarding fracture history over the past 5 years. All reported fractures were radiographically confirmed. |
| Outcomes | - To investigate the utility of FRAX and Garvan tool in daily practice |

| **Study** | **Evaluation of different screening tools for predicting femoral neck osteoporosis in rural South Indian postmenopausal women**  **Cherian 2018** |
| --- | --- |
| Study type | Cross sectional study |
| Number of studies/ number of participants | N= 2108 |
| Countries and Settings | South India |
| Funding | Not reported |
| Duration of study | Between 2014 and 2016 |
| Age, gender, ethnicity, baseline fracture | Age [mean (range)]: 50 years and above  Gender (n. F): Intervention group: 100%; Placebo group: 100%  Baseline fracture: 126 (6%) |
| Patient characteristics | All ambulatory rural postmenopausal women aged 50 years and above were recruited from the Vellone district of southern India.  Women with a prior diagnosis of osteoporosis, malignancy, stroke, or other conditions leading to immobilization, chronic kidney disease, and chronic liver disease were excluded. Those women on treatment with bisphosphonates and anabolic agents were also excluded |
| Intervention | The subjects were classified as osteoporosis, osteopenia, and normal, depending on the World Health Organization T-score of ≤-2.5, -2.5 to -1.0, and normal ≥-1, respectively.  In the present study, FRAX was used without BMD |
| Outcomes | - To assess the diagnostic performance of 6 internationally validated tools for the diagnosis of osteoporosis at the femoral neck |

| **Study** | **Evaluation of the validity of osteoporosis and fracture risk assessment tools (IOF One Minute Test, SCORE, and FRAX) in postmenopausal Palestinian women**  **Kharroubi 2017** |
| --- | --- |
| Study type | Cross sectional study |
| Number of studies/ number of participants | N= 382 |
| Countries and Settings | West Bank region of Palestine |
| Funding | Not reported |
| Duration of study | Between 1997 and 2008 |
| Age, gender, ethnicity | Age [mean (range)]: 45 years and above  Gender (n. F): Intervention group:100%; Placebo group: 100% |
| Patient characteristics | All recruited subjects were not previously diagnosed with bone problems or suffered from bone-related health complications. None of the subjects were using any prescription drugs or food supplements (including vitamin D and calcium) that might affect their general bone status |
| Intervention | In this study the FRAX was calculated without BMD |
| Outcomes | - To evaluate the validity of the updated IOF One Minute Osteoporosis Risk Assessment Test, FRAX, SCORE as well as age alone to detect the risk of developing osteoporosis in postmenopausal Palestinian women |

| **Study** | **FRAX based intervention thresholds for management of osteoporosis in Singaporean women**  **Chandran 2018** |
| --- | --- |
| Study type | Retrospective review |
| Number of studies/ number of participants | N= 1001 |
| Countries and Settings | Singapore General Hospital |
| Funding | Not reported |
| Duration of study | Between 2014 and 2017 |
| Age, gender, ethnicity | Age [mean (range)]: 50 years and above  Gender (n. F): Intervention group:100%; Placebo group: 100%  Baseline fracture: 60 (6%) |
| Patient characteristics | Subjects were excluded from the study if they were premenopausal or had ever been treated for osteoporosis. Subjects who had incomplete baseline socio-demographic information, medical, menstrual, fracture, smoking, alcohol and medical history and laboratory data were also excluded from the final analysis as were subjects with uninterpretable DXA scans of the hip and lumbar vertebrae |
| Intervention | In this study were used ethnic-specific Singapore FRAX models |
| Outcomes | - To explore FRAX-based intervention thresholds that could potentially be considered for the management of osteoporosis in postmenopausal Singaporean women |

| **Study** | **Increased cortical porosity and reduced cortical thickness of the proximal femur are associated with nonvertebral fracture independent of Fracture Risk Assessment Tool and Garvan estimates in postmenopausal women**  **Kral 2017** |
| --- | --- |
| Study type | Nested case-control study |
| Number of studies/ number of participants | N= 443 |
| Countries and Settings | Tromsø Study in Norway |
| Funding | The North Norwegian Health Authorities funded the study |
| Duration of study | Between 1994 and 1995 |
| Age, gender, ethnicity, baseline fracture | Age [mean (range)]: 50 years and above  Gender (n. F): Intervention group:100%; Placebo group: 100%  Baseline fracture: 61 (14%) |
| Patient characteristics | The study included women that suffered at least one fracture of the hip, wrist, or proximal humerus after the age of 50 years.  Those who were premenopausal, received bisphosphonates, or had hip prostheses or metal screws in the hip region were excluded from the study |
| Intervention | This study used the online country-specific FAX algorithm for Norway |
| Outcomes | - To test the hypothesis that cortical parameters are associated with fracture risk, independent of FRAX ad Garvan estimates |

| **Study** | **Osteoporosis screening in postmenopausal women 50 to 64 years old: comparison of US preventive services task force strategy and two traditional strategies in the women’s health initiative**  **Crandall 2014** |
| --- | --- |
| Study type | Retrospective cohort study |
| Number of studies/ number of participants | N= 5165 |
| Countries and Settings | 40 clinical centers (Tucson and Phoenix, AZ; Pittsburgh, PA; and Birmingham, AL) |
| Funding | The WHI program is funded by the National Heart, Lung, and Blood Institute, national Institutes of Health, US Department of Health and Human Services |
| Duration of study | Not reported |
| Age, gender, ethnicity, baseline fracture | Age [mean (range)]: 50 – 79 years  Gender (n. F): Intervention group:100%; Placebo group: 100%  Baseline fracture: 62 (1%) |
| Patient characteristics | Eligibility criteria included being aged 50-79 years at baseline, postmenopausal, and free from serious medical conditions. All WHI participants were postmenopausal, defined as at least 6 months of amenorrhea for women ≥ 55 years, and at least 12 months of amenorrhea for women aged 50 to 54 years. They were also not taking medications known to influence BMD and for whom information regarding femoral neck T-score and osteoporosis risk factors was complete |
| Intervention | This study used the US Preventive Services Task Force (USPSTF) FRAX without BMD |
| Outcomes | - The proportion of women for whom BMD testing would have been recommended according to each of the three risk-assessment strategies overall, and classified by femoral neck T-score category - The proportion of women with femoral neck T-score ≤-2.5 who would be identified for screening under each strategy - The sensitivity, specificity, and area under the receiver operating characteristic curve (AUC) for identifying low BMD and osteoporosis under each strategy - To calculate the AUC of the three tools for identifying of T-score ≤-2.5 at one or more of the following sites: lumbar spine, total hip, or femoral neck - To estimate the cut-off score that would identify 90% of women with femoral neck T-score ≤-2.5 |

| **Study** | **Possible FRAX-based intervention thresholds for a cohort of Chinese postmenopausal women**  **Cheung 2014** |
| --- | --- |
| Study type | Prospective population-based study |
| Number of studies/ number of participants | N= 2266 |
| Countries and Settings | Part of the Hong Kong Osteoporosis Study |
| Funding | Not reported |
| Duration of study | Between 1995 and 2009 |
| Age, gender, ethnicity, baseline fracture | Age [mean (range)]: 40 years and above  Gender (n. F): Intervention group:100%; Placebo group: 100%  Baseline fracture: 291 (13%) |
| Patient characteristics | Subjects who had already been prescribed osteoporosis treatment were excluded. At least one year has passed between the last menstrual cycle and the baseline assessment for all subjects |
| Intervention | Patients were followed up yearly by structured telephone interview to assess the occurrence of MOFs of the wrist, clinical spine, hip or humerus. Reports of fractures were subsequently confirmed using the subjects’ medical records, which were readily accessible using the centrally linked, computerized network of the Hong Kong Hospital Authority |
| Outcomes | - To determine the impact of applying different intervention thresholds to a cohort of Chinese postmenopausal women |

| **Study** | **Setting the new FRAX reference threshold without bone mineral density in Chinese postmenopausal women**  **Liu 2020** |
| --- | --- |
| Study type | Retrospective cohort study |
| Number of studies/ number of participants | N= 264 |
| Countries and Settings | Community medical centers in Changsha City, Hunan Province, China |
| Funding | The Natinal Nature Science Foundation of China, the Hunan Nature Science Foundation, and Bethune Charitable Foundation |
| Duration of study | Between 2017 and 2008 |
| Age, gender, ethnicity, baseline fracture | Age [mean (range)]: 50 years and above  Gender (n. F): Intervention group:100%; Placebo group: 100%  Baseline fracture: 21 (8%) |
| Patient characteristics | The study included postmenopausal women aged over 50 years.  The only exclusion criterion was a history of anti-osteoporotic medication |
| Intervention | All the participants completed the standard medical assessment questionnaires by themselves.  The study used the FRAX model (modified Chinese version) |
| Outcomes | - To explore the Chinese-specific thresholds of FRAX without the T-score |

| **Study** | **The discriminative ability of FRAX, the WHO algorithm, to identify women with prevalent asymptomatic vertebral fractures: a cross-sectional study**  **El Maghraoui 2014** |
| --- | --- |
| Study type | Cross- sectional study |
| Number of studies/ number of participants | N= 908 |
| Countries and Settings | Cracow Medical Centre |
| Funding | Not reported |
| Duration of study | Between 2010 and 2012 |
| Age, gender, ethnicity | Age [mean (range)]: 50 years and above  Gender (n. F): Intervention group:100%; Placebo group: 100%  Baseline fracture: 382 (42%) |
| Patient characteristics | 908 consecutive women who had no previous diagnosis of osteoporosis were entered into the study.  General exclusion criteria were non-Caucasian origin and diseases, drugs, and other major determinants known to affect bone metabolism. There were also excluded subjects with gastrectomy, intestinal resection, recent hyperthyroidism or hyperparathyroidism, recent severe immobilization, treatment with corticosteroids, breast cancer or aromatase inhibitors. |
| Intervention | Each subject completed a standardized questionnaire designed to document putative risk factors of osteoporosis.  The study used FRAX with and without BMD |
| Outcomes | - To assess the predictive value of FRAX tool - To assess the prevalence of clinical risk factors and, in particular, of osteoporotic fractures |

| **Study** | **Validation of osteoporosis risk assessment tools in middle-aged Thai women**  **Indhavivadhana 2016** |
| --- | --- |
| Study type | Retrospective study |
| Number of studies/ number of participants | N= 1038 |
| Countries and Settings | Siriraj Menopause Clinic, Siriraj Hospital, a tertiary-care hospital of Mahidol University, Thailand |
| Funding | Nil |
| Duration of study | Between 1997 and 2006 |
| Age, gender, ethnicity | Age [mean (range)]: 40 - 60 years  Gender (n. F): Intervention group:100%; Placebo group: 100% |
| Patient characteristics | The study involved women in perimenopause, natural postmenopause, surgical menopause, and premature menopause |
| Intervention | The patients were classified as having osteoporosis, osteopenia, or normal. The study used FRAX without BMD |
| Outcomes | - To validate osteoporosis risk assessments tools in middle-aged Thai women |

| **Study** | **Validation of the FRAX predictive model for major osteoporotic fracture in a historical cohort of Spanish women**  **Tebé CordomÍ 2013** |
| --- | --- |
| Study type | Retrospective cohort study |
| Number of studies/ number of participants | N= 1231 |
| Countries and Settings | CETIR database (CDB) |
| Funding | This study was funded by the Plan de Calidad para el Sistema Nacional de Salud in collaboration with the Instituto Carlos III and the Agència d’Informació, Avaluació i Qualitat en Salut |
| Duration of study | Between 1992 and 2008 |
| Age, gender, ethnicity, baseline fracture | Age [mean (range)]: 40-90 years  Gender (n. F): Intervention group:100%; Placebo group: 100%  Baseline fracture: 185 (15%) |
| Patient characteristics | The study involved women aged 40-90 years with a first visit or a bone densitometry |
| Intervention | An interview-led questionnaire was administered by trained technicians at first visits and subsequent follow-ups. |
| Outcomes | - To assess the predictive ability of the Spanish FRAX for major osteoporotic fracture in women with basal BMD measurements and 10-yr follow-up |

| **Study** | **Fracture Risk Assessment With FRAX Using Real-World Data in a Population Based Cohort From Israel**  **Goldshtein 2018** |
| --- | --- |
| Study type | Population-based cohort study |
| Number of studies/ number of participants | N= 141,320 women |
| Countries and Settings | Maccabi Healthcare Services (MHS), a large Israeli government-funded health maintenance organization (HMO) |
| Funding | Not reported |
| Duration of study | Recruitment: 2004 – 2006 + follow-up |
| Age, gender, ethnicity | Without major osteoporotic fractures (MOF) n. 122280, mean age 58.0 (53.0 – 66.0)  With MOF n. 19040, mean age 65.0 (57.0 – 74.0)  Baseline fracture: 6089 (4.3%) |
| Patient characteristics | A total of 141,320 female MHS members were eligible (denoted the “total population”), out of which 16,578 patients had an electronically available BMD test performed before June 2006 |
| Intervention | Data on diagnosis codes, medication dispensations, and demographic factors were extracted from the EMRs to populate the clinical risk factors used in FRAX. |
| Outcomes | The 2 endpoint events were incident hip (femoral neck) fracture and incident major osteoporotic fracture (MOF) during the 10-year follow up period, including fractures of the femoral neck, clinical spine, forearm, and proximal humerus, in accordance with FRAX definitions |

| **Study** | **Clinical risk factors for osteoporosis in Ireland and the UK: a comparison of FRAX and QFractureScores**  **Cummins 2011** |
| --- | --- |
| Study type | Case-control study |
| Number of studies/ number of participants | N= 246 |
| Countries and Settings | UK, Ireland |
| Funding | This study was supported by the Enterprise Ireland Innovation Partnership grant board |
| Duration of study | Between 1992 and 1994 |
| Age, gender, ethnicity | Age [mean (range)]: 50-85 years  Gender (n. F): Intervention group:100%; Placebo group: 100%  Baseline fracture: 246 (100%) |
| Patient characteristics | The study involved Caucasian women aged 50-85 years who were at least postmenopausal. Participants included subjects who had recently suffered a fracture as well as individual who had never suffered a fracture.  Subjects who were receiving treatment for osteoporosis, those on corticosteroids, and those with a secondary cause of osteoporosis such as malabsorption, chronic liver disease, renal failure, and malignant disease were excluded |
| Intervention | FRAX scores were calculated manually from the FRAX Web site, with double data entry in 10% of subjects. The UK version of FRAX was used for all subjects as an Irish version of FRAX is not currently available.  The 10-year probabilities of major osteoporotic and hip fracture with and without BMD were recorded for FRAX. |
| Outcomes | - To compare the performance of FRAX and QFracture algorithms in identifying patients who suffered fractures |
| **Study** | **Predicting fractures in an international cohort using risk factor algorithms, without bone mineral density**  **Sambrook 2011** |
| Study type | Prospective cohort study |
| Number of studies/ number of participants | N= 19586 |
| Countries and Settings | GLOW study |
| Funding | Financial support for the GLOW study is provided by Warner Chilcott Company, LLC and Sanofi-aventis to the Center for Outcomes Research, university of Massachusetts medical School |
| Duration of study | Between 2008 and 2010 |
| Age, gender, ethnicity | Age [mean (range)]: 60 years and above  Gender (n. F): Intervention group:100%; Placebo group: 100%  Baseline fracture: 4008 (20.4%) |
| Patient characteristics | The study involved women aged 55 years and older who had been attended by their physician in the past 24 months.  Patients were excluded if they were unable to complete the study survey due to cognitive impairment, language barriers, institutionalization, or illness. |
| Intervention | A Baseline questionnaires, along with invitations to participate in the study signed by the local principal investigator, were mailed to all potential subjects. non-respondents were followed up with sequential postcard reminders, second questionnaires, and telephone interviews. Questionnaires were mailed at 1 and 2 years to determine incident fractures. |
| Outcomes | - To investigate the utility of FRAX and Garvan tool in daily practice |

| **Study** | **Comparison of three tools for predicting primary osteoporosis in an elderly male population in Beijing: a cross-sectional study**  **Zhang 2018** |
| --- | --- |
| Study type | Cross-sectional study |
| Number of studies/ number of participants | N= 1349 |
| Countries and Settings | Beijing Friendship Hospital, Capital Medical University. |
| Funding | Not reported |
| Duration of study | 2014 - |
| Age, gender, ethnicity, baseline fracture | Mean age 65.2 ± 8.68 (Range 50-90)  Ethnicity: Chinese  Baseline fracture: 115 (8.5%) |
| Patient characteristics | Chinese men aged 50 years consecutively recruited from three community health service centers (Fangzhuang, Tuanjiehu, and Wangzuo) from January 2014 to October 2015. |
| Intervention | three clinical tools, the Osteoporosis Self-Assessment Tool for Asians (OSTA), Fracture Risk Assessment Tool (FRAX) without bone mineral density (BMD), and body mass index (BMI), for predicting primary osteoporosis (OP) were compared. |
| Outcomes | Primary osteoporosis according to the WHO at lumbar spine, worst hip, femoral neck, and total hip site. |

| **Study** | **FRAX calculator and Garvan nomogram in male osteoporotic population**  **Pluskiewicz 2014** |
| --- | --- |
| Study type | Cross-sectional study. |
| Number of studies/ number of participants | N= 801 |
| Countries and Settings | Poland |
| Funding | Not reported |
| Duration of study | Recruitment: 2009 – 2010. Follow.up not reported |
| Age, gender, ethnicity | mean age 70.8 ± 9.31  ethnicity: polish  baseline fracture: 218 (27.2%) |
| Patient characteristics | The studied group included 801 men evaluated at four osteoporotic outpatient clinics in four different centers. |
| Intervention | The 10-year fracture prediction was established, using the FRAX calculator and Garvan nomogram. |
| Outcomes | ‘‘major fractures", in general confined to hip, humerus, spine and wrist fro FRAX, and ‘‘all fractures’’ for Garvan, whose category is much broader and includes more fracture sites. |

| **Study** | **Validation of three tools for identifying painful new osteoporotic vertebral fractures in older Chinese men: bone mineral density, Osteoporosis Self-Assessment Tool for Asians, and fracture risk assessment tool**  **Lin 2016** |
| --- | --- |
| Study type | Cross-sectional study. |
| Number of studies/ number of participants | N= 496 |
| Countries and Settings | Osteoporosis Clinic at Beijing Friendship Hospital |
| Funding | Not reported |
| Duration of study | 2013-2015 |
| Age, gender, ethnicity, baseline fracture | Age, gender, ethnicity: Not reported  Baseline fracture: 120 (24.2%) |
| Patient characteristics | Men aged 50 years were apportioned to a group for men with fractures who had undergone percutaneous vertebroplasty (n=111), or a control group of healthy men (n=385). |
| Intervention | Three tools for predicting painful new osteoporotic vertebral fractures (PNOVFs) in older Chinese men: bone mineral density (BMD), the Osteoporosis Self-Assessment Tool for Asians (OSTA), and the World Health Organization fracture risk assessment tool (FRAX) (without BMD). |
| Outcomes | Diagnosis of osteoporosis was determined by a BMD T-score of #2.5 standard deviations below the average for a young adult at peak bone density at the femoral neck, total hip, or L1–L4. |

| **Study** | **Fracture risk assessment in postmenopausal women with diabetes: comparison between DeFRA and FRAX tools**  **Bonaccorsi 2017** |
| --- | --- |
| Study type | Case-control study |
| Number of studies/ number of participants | N= 237 |
| Countries and Settings | Osteoporosis Centre of University of Ferrara Italy |
| Funding | Not reported |
| Duration of study | 2015 |
| Age, gender, ethnicity | Age [mean (range)]: 50 years and above  Gender (n. F): Intervention group:100%; Placebo group: 100%  Baseline fracture: 9 (3.8%) |
| Patient characteristics | The study involved women in postmenopausal status according to Straw classification; age between 40 and 90 years, body mass index < 37kg/m^2^ which is the upper limit for TBS calculation; preserved renal function.  The a priori exclusion criteria were: treatment with drugs that may interfere with mineral and bone metabolism for the last 2 years; relevant comorbidities |
| Intervention | Risk fracture was assessed using the FRAX tool for Europe-Italy, and included those CRFs for osteoporosis as collected in the patient’s interview together with femoral neck T-score.  In order to assess the 10-year fracture risk probability with the DeFRA tool, the lowest T-score between proximal femur and lumbar spine was used together with several CRFs. |
| Outcomes | - To compare the performance of Fracture Risk Assessment Tool (FRAX) with that of Derived FRAX (DeFRA) in estimating fracture risk in a cohort of type-2 diabetes mellitus postmenopausal women - - To investigate the clinical and morphometric vertebral fractures and self-reported history of non-vertebral FFs, namely hip, humerus, and radius |

| **Study** | **Probability of fractures predicted by FRAX® and observed incidence in the Spanish ECOSAP Study cohort**  **González-Macías 2012** |
| --- | --- |
| Study type | A prospective cohort study, by a nonrandomized sampling |
| Number of studies/ number of participants | N=5201 |
| Countries and Settings | 58 primary care centers of the National Health Service (NHS) throughout Spain |
| Funding | This study was supported by an unrestrictive research grant from the Medical Research Department, Eli Lilly and Company. Madrid. Spain. |
| Duration of study | Patient recruited between March 2000 and June 2001; Duration of the study: 3 years |
| Age, gender, ethnicity, baseline fracture | Age [mean (SD)]: 52.3 (5.3)  Gender (% F): 100%  Ethnicity (% Caucasian): 100%  Baseline fracture: 20.2% |
| Patient characteristics | The study comprised a total of 5201 **Caucasian women** aged 65 or older, recruited in 58 primary care centers of the National Health Service (NHS) throughout Spain between March 2000 and June 2001, regardless of the reason for consultation. Given the characteristics of the medical care provided by the Spanish NHS, the ECOSAP Study cohort is considered representative of the general population of Spanish women of that age group. Only low-energy trauma fractures, defined as secondary to minor trauma or a fall from the standing position to floor level, were analyzed. Pathological fractures were excluded, as were those caused by severe trauma (traffic accidents, impact of moving objects, falling from greater than standing height) and fractures of the skull, face, metacarpals and phalanges. |
| Intervention | The individual 10-year absolute risks of hip and MOF were calculated with the FRAX® algorithms for Spain without the inclusion of the bone mineral density (BMD) measurements. Calibration was evaluated by comparing the three-year estimated (E) fractures predicted with FRAX® with the number of observed (O) fractures, and their discriminative ability for the probability of new fractures with the area under the receiving operating characteristic (ROC) curves. |
| Outcomes | The 10-year fracture probabilities calculated with the FRAX® tool were annualized to extrapolate the 3-year fracture probabilities, assuming a linear fracture risk over time. The ability of the FRAX algorithms to discriminate those women who will develop a fracture and those who will not, was measured as the areas under the receiver operating characteristic (ROC) curves (AUC). |

| **Study** | **The Fracture and Immobilization Score (FRISC) for risk assessment of osteoporotic fracture and immobilization in postmenopausal women―A joint analysis of the Nagano, Miyama, and Taiji Cohorts**  **Tanaka 2010** |
| --- | --- |
| Study type | A community-based cohort study |
| Number of studies/ number of participants | The Nagano Cohort N=1787, The Miyama and Taiji Cohorts (N=400) |
| Countries and Settings | The authors used two independent datasets in the current analysis; a developmental dataset from the Nagano Cohort and a validation dataset from the Miyama and Taiji Cohorts. |
| Funding | The Miyama Cohort was set up in 1988 as subsets of nationwide community-based cohort studies sponsored by the Ministry of Education or Ministry of Health and Welfare |
| Duration of study | Median follow-up of 5.3 years |
| Age, gender, ethnicity, baseline fracture | Age [mean (SD)]: The Nagano Cohort: 63.4 (11.1), The Miyama and Taiji Cohorts: 59.5 (11.3)  Gender (% F): 100%  Ethnicity (% Japanese): 100%  Baseline fracture: The Nagano Cohort: 403 (22.6%), The Miyama and Taiji Cohorts: 49 (25%) |
| Patient characteristics | - The Nagano Cohort recruited and followed up postmenopausal women who were receiving medical care as outpatients or visitors at a medical institute in Nagano Prefecture, Japan since April 1993. A total of 1787 participants were included in the developmental dataset; exclusion criteria were (i) metabolic bone disease and (ii) secondary osteoporosis (e.g. hyperparathyroidism, hyperthyroidism other than patients on T4 replacement and with euthyroid for more than one year, chronic renal failure or osteomalacia). - The Miyama Cohort was set up in 1988 as subsets of nationwide community-based cohort studies sponsored by the Ministry of Education or Ministry of Health and Welfare. A total of 1453 inhabitants aged 40–79 years in Miyama Village were listed from the resident registration in December 1988. Then, 200 men and 200 women were recruited and followed up between 1990 and 2000. - The Taiji Cohort is a community-based cohort study in Taiji Town, Wakayama Prefecture, Japan [25–27]. From a list of 2261 inhabitants aged 40–79 years obtained from the resident registration in June 1992, 50 men and 50 women in each decade age group between 40 and 79 years (a total of 400 participants) were recruited randomly and followed up between 1993 and 2003. |
| Intervention | The following variables were initially identified from the literature as the traditional risk factors for osteoporotic fracture: covariates included in the FRAX other than femoral neck BMD (age, height, weight, prior fracture, parental history of fracture, current smoking status, use of steroids, rheumatoid arthritis, alcohol intake), lumbar BMD, presence of back pain, presence of any pain, and drug treatment for osteoporosis |
| Outcomes | Endpoints included the annual incidence of major osteoporotic fracture and immobilization. The authors assessed the predictive accuracy of the FRISC in terms of calibration and discrimination using occurrence of MOF within a 10-year period, which was treated as a binary event, in the validation dataset. Calibration, namely how closely the prediction reflects actual events, was assessed using ratio of observed and predicted events and the Hosmer–Lemehshow test. Discrimination, the ability to distinguish between those who experience the event and those who do not, was evaluated using receiver operating characteristic (ROC) curves and Harrell's C statistic. |

# Diagnostic Accuracy

## Sensitivity and Specificity, FRAX

### **WOMEN**

**MOF without BMD at 3%**

**
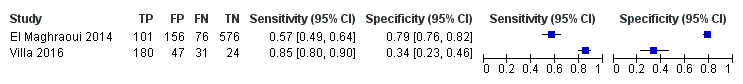
**

**MOF without BMD at 5%**

**
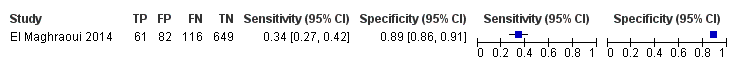
**

**MOF without BMD at 10%**

**
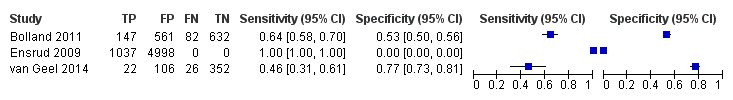
**

**MOF without BMD at 20%**

**
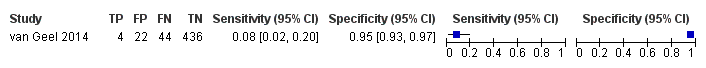
**

**MOF without BMD at 30%**

**
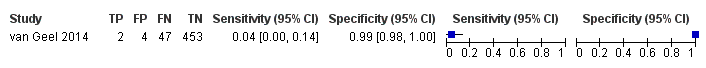
**

**MOF with BMD at 3%**

**
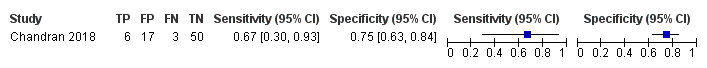
**

**MOF with BMD at 5%**

**
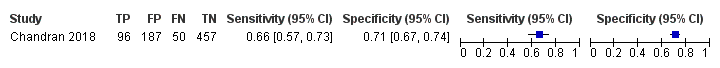
**

**MOF with BMD at 10%**

**
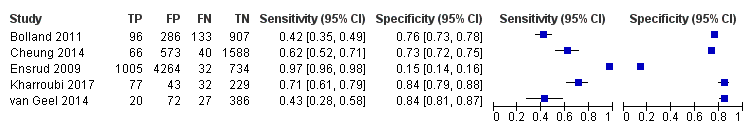
**

**MOF with BMD at 20%**

**
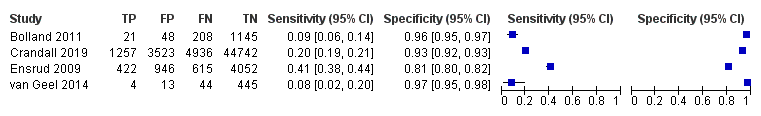
**

**MOF with BMD at 30%**

**
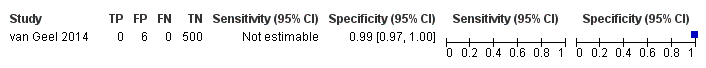
**

**HIP without BMD at 3%**

**
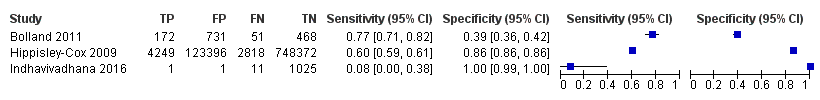
**

**HIP without BMD at 5%**

**
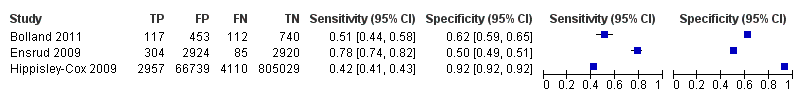
**

**HIP with BMD at 3%**

**
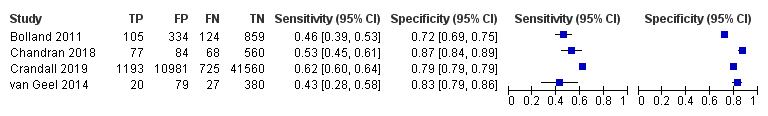
**

**HIP with BMD at 5%**

**
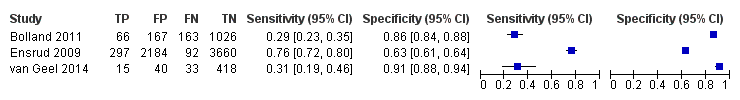
**

**HIP with BMD at 10%**

**
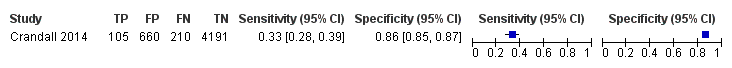
**

### **POPULATION**

**MOF without BMD at 3%**

**
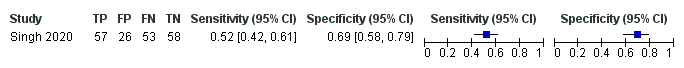
**

**MOF without BMD at 5%**

**
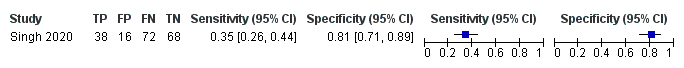
**

**MOF without BMD at 10%**

**
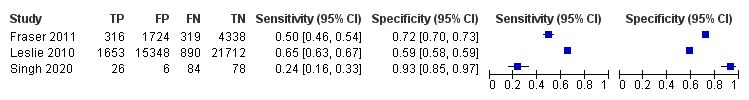
**

**MOF without BMD at 20%**

**
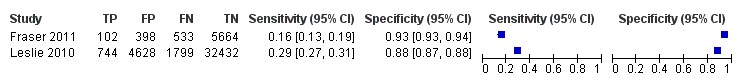
**

**MOF without BMD at 30%**

**
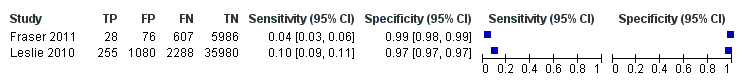
**

**MOF with BMD at 10%**

**
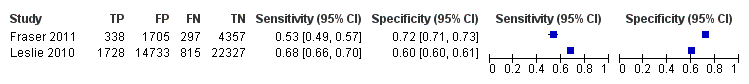
**

**MOF with BMD at 20%**

**
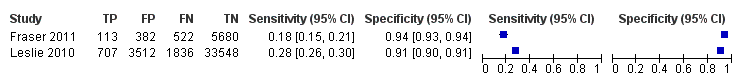
**

**MOF with BMD at 30%**

**
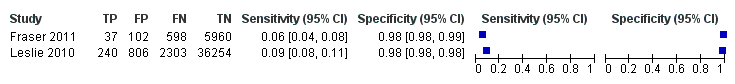
**

**HIP without BMD at 3%**

**
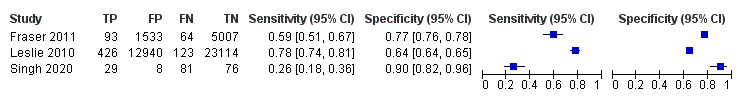
**

**HIP without BMD at 5%**

**
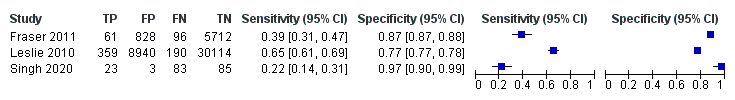
**

**HIP with BMD at 3%**

**
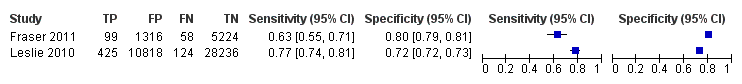
**

**HIP with BMD at 5%**

**
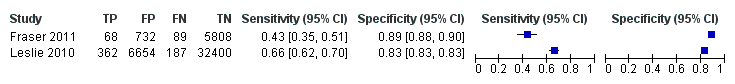
**

### **WOMEN and POPULATION**

**MOF without BMD at 3%**

**
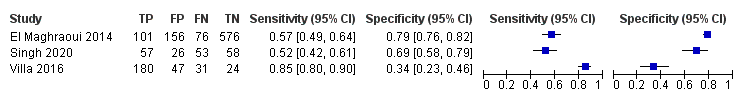
**

**MOF without BMD at 5%**

**
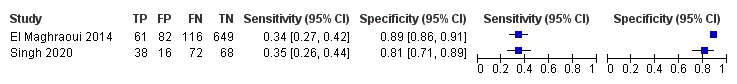
**

**MOF without BMD at 10%**

**
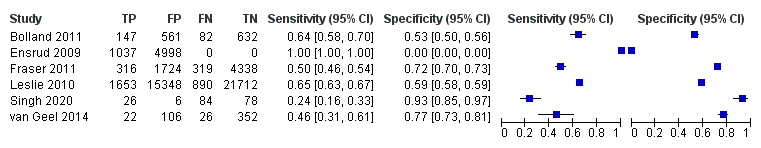
**

**MOF without BMD at 20%**

**
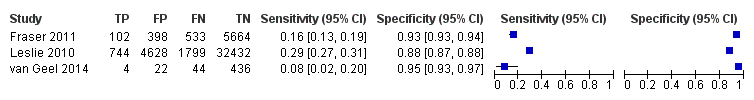
**

**MOF without BMD at 30%**

**
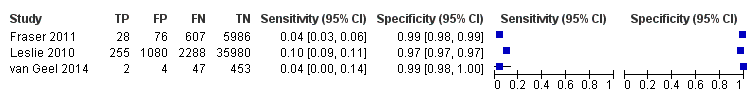
**

**MOF with BMD at 10%**

**
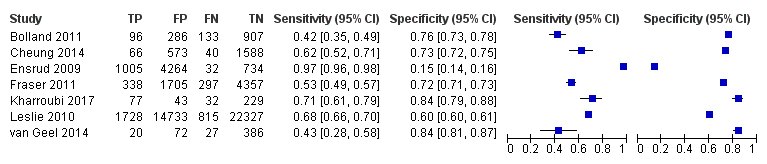
**

**MOF with BMD at 20%**

**
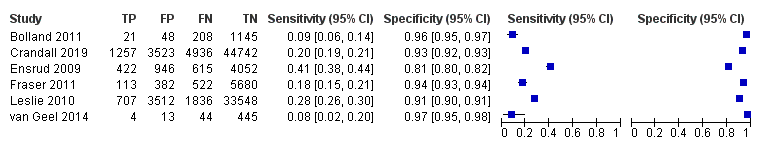
**

**MOF with BMD at 30%**

**
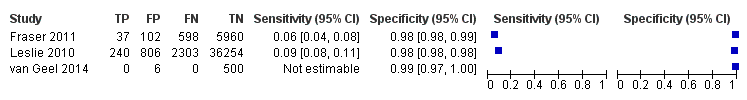
**

**HIP without BMD at 3%**

**
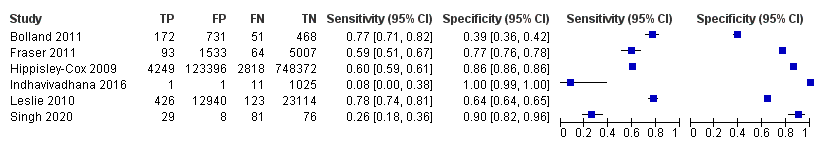
**

**HIP without BMD at 5%**

**
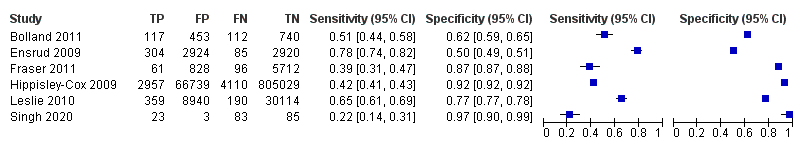
**

**HIP with BMD at 3%**

**
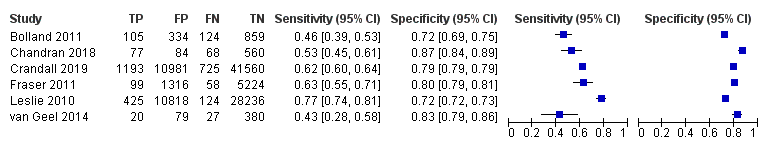
**

**HIP with BMD at 5%**


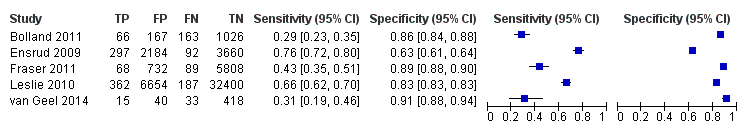


## Area under the curve, FRAX

### **WOMEN**

**MOF with BMD**


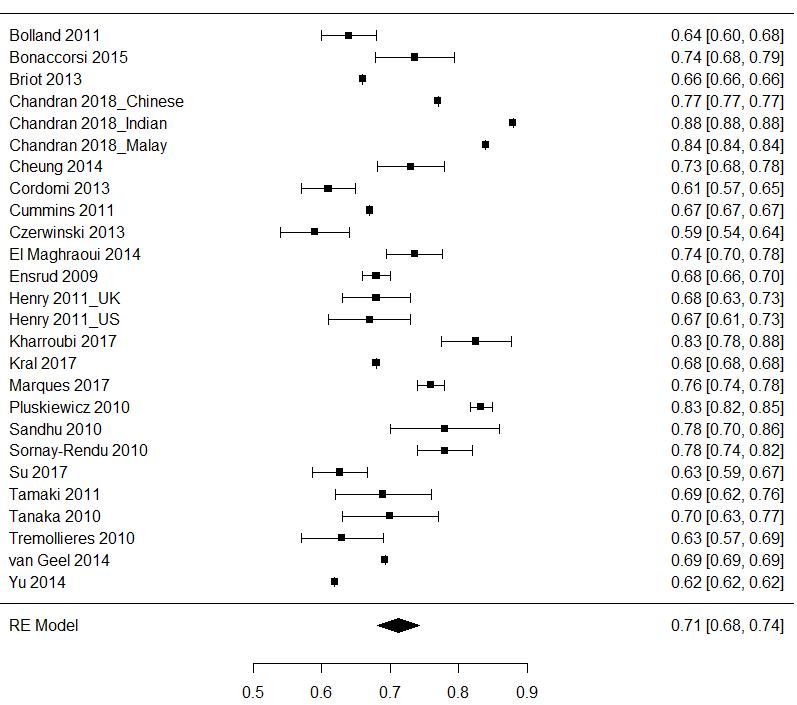


**MOF without BMD**


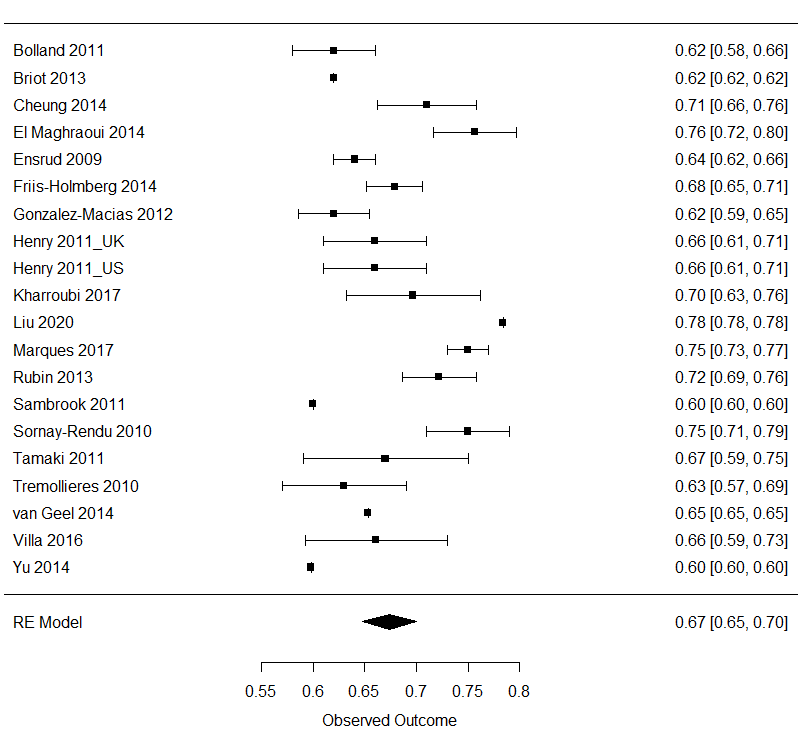


**HIP with BMD**


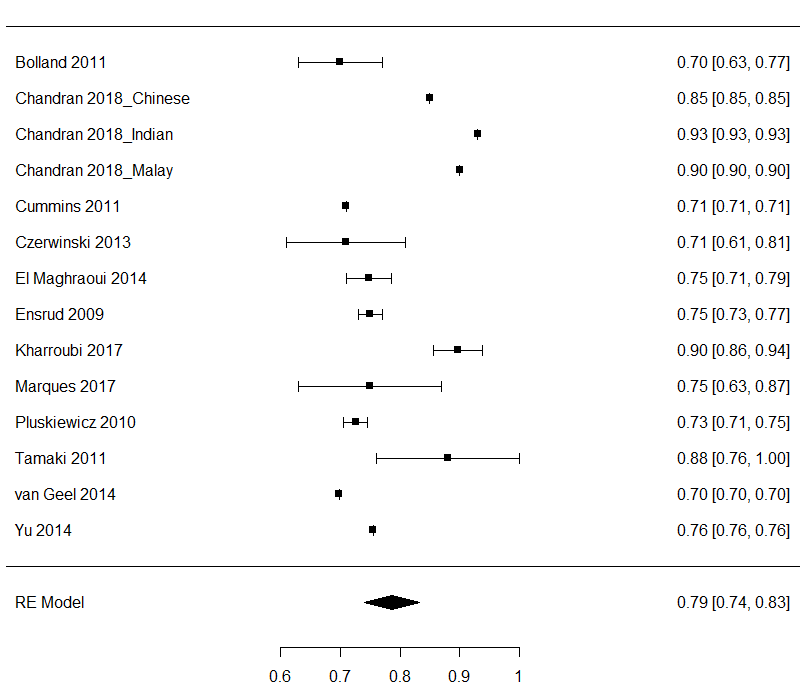


**HIP without BMD**


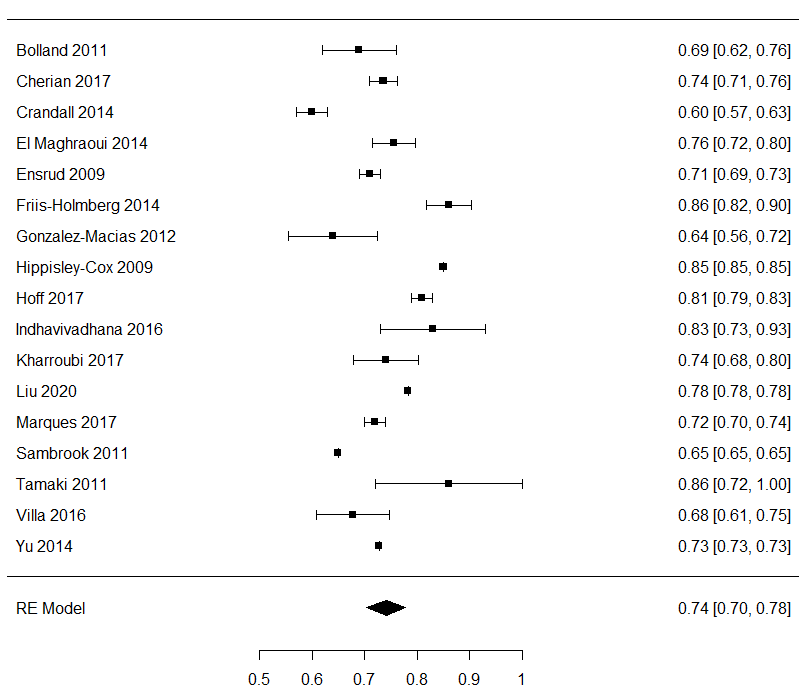


### **MEN**

**MOF with BMD**


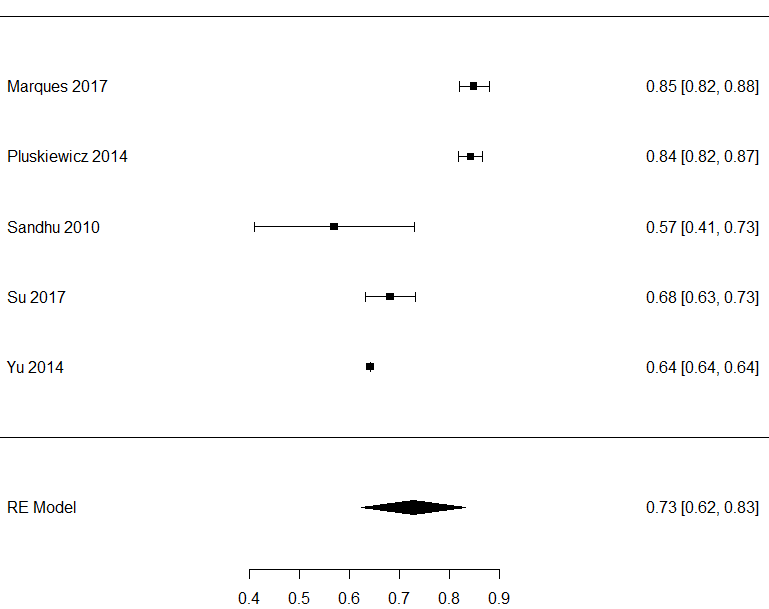


**MOF without BMD**


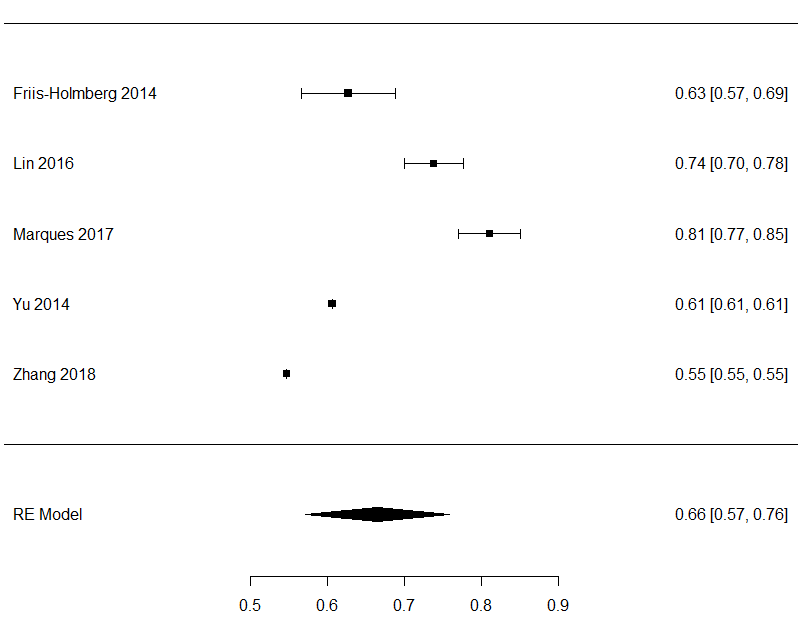


**HIP with BMD**


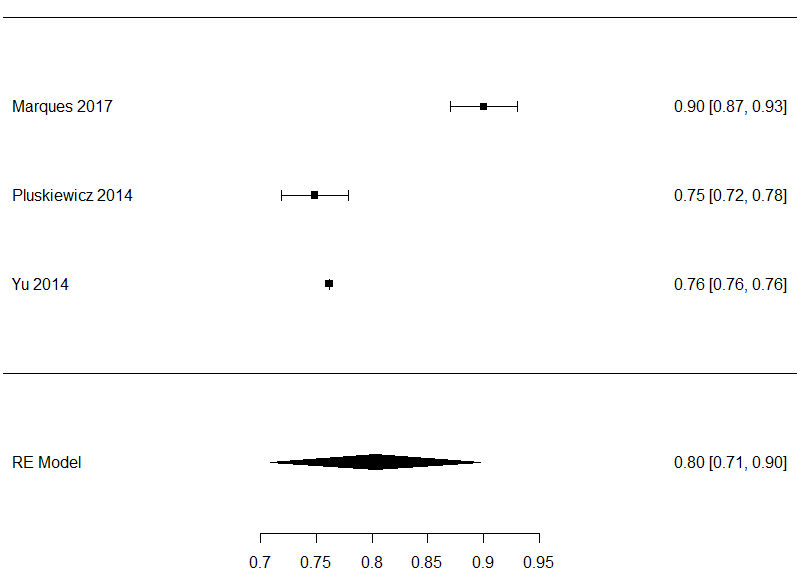


**HIP without BMD**


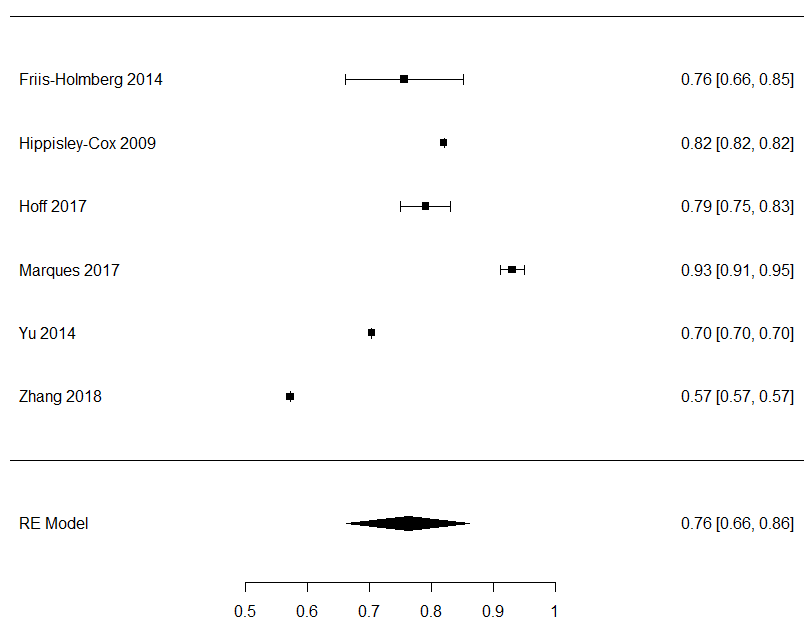


### **POPULATION**

**MOF with BMD**


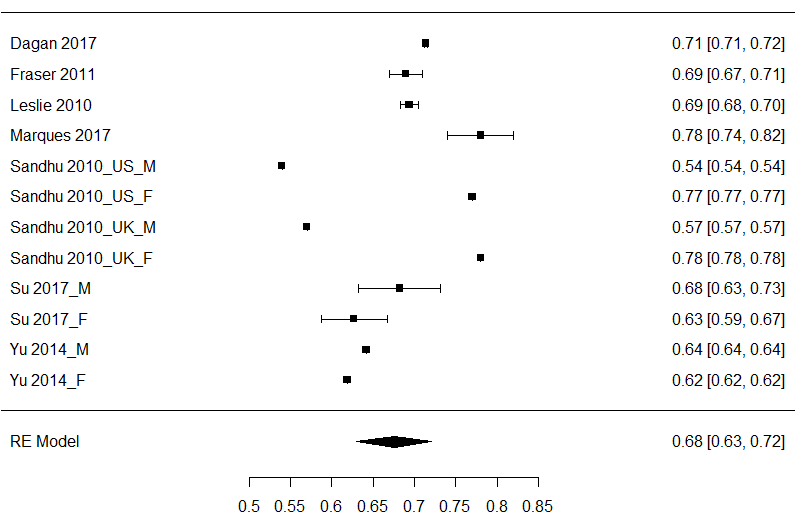


**MOF without BMD**


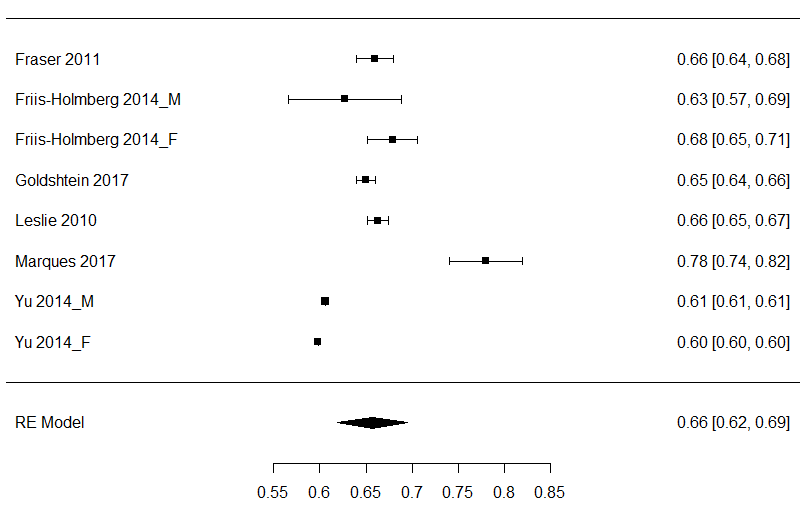


**HIP with BMD**


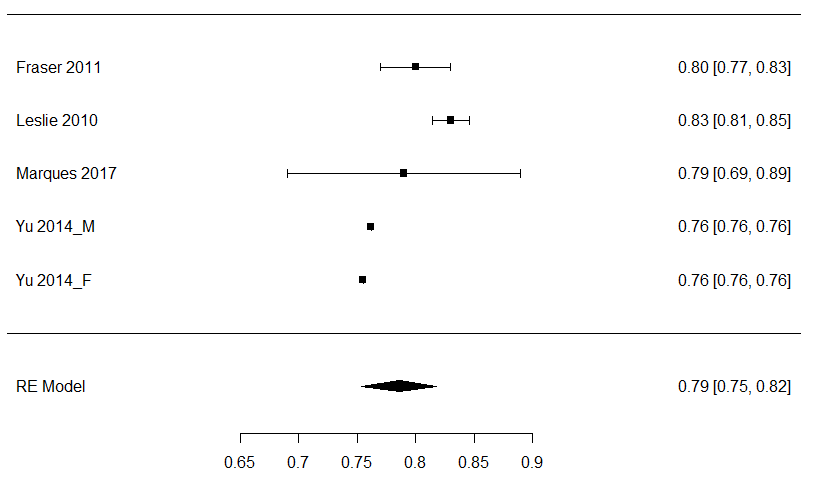


**HIP without BMD**


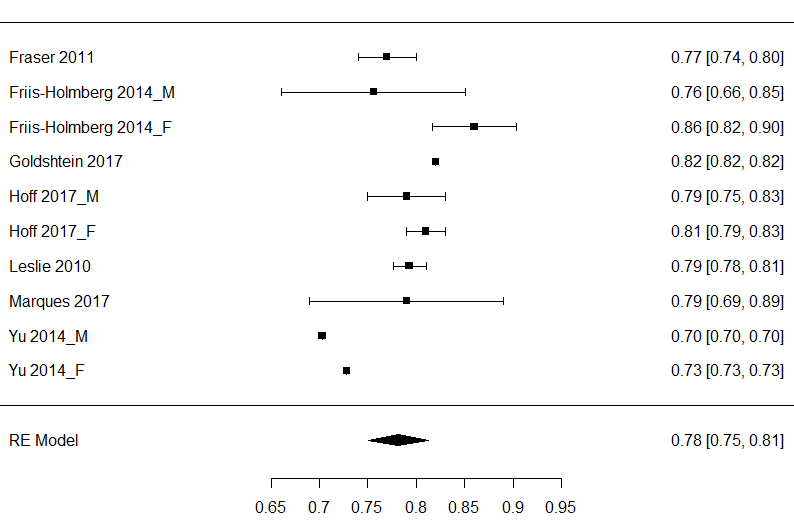


### **WOMEN, MEN AND POPULATION**

**MOF with BMD**


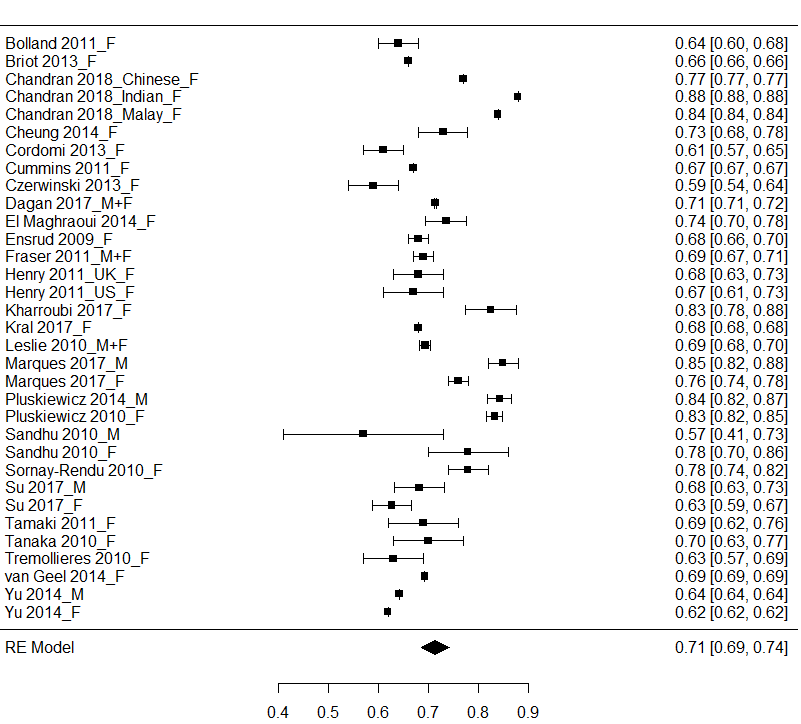


**MOF without BMD**


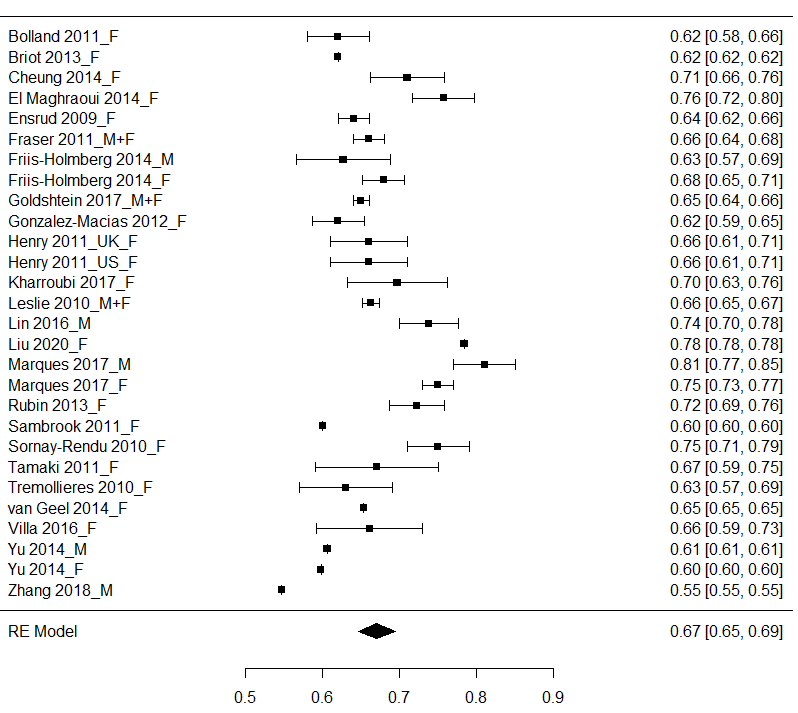


**HIP with BMD**


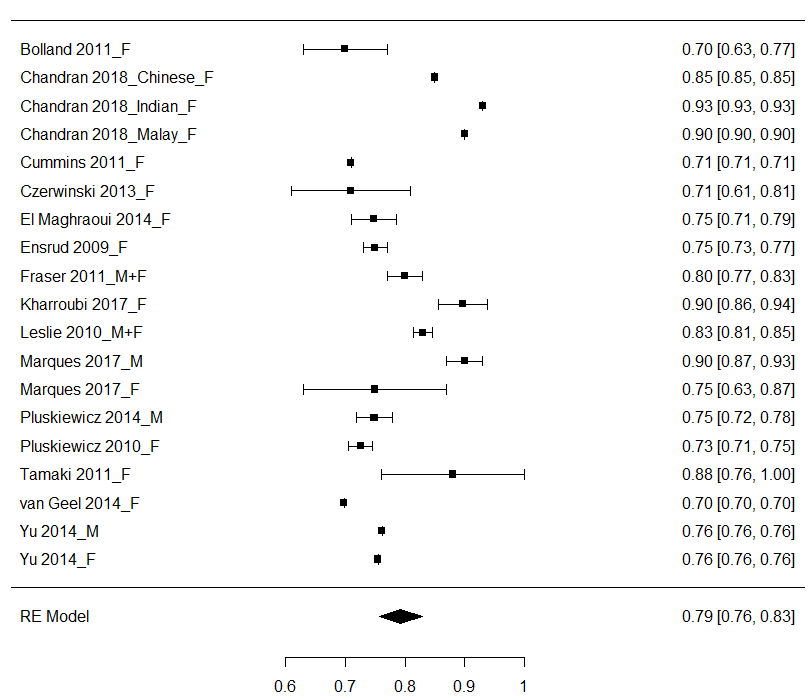


**HIP without BMD**


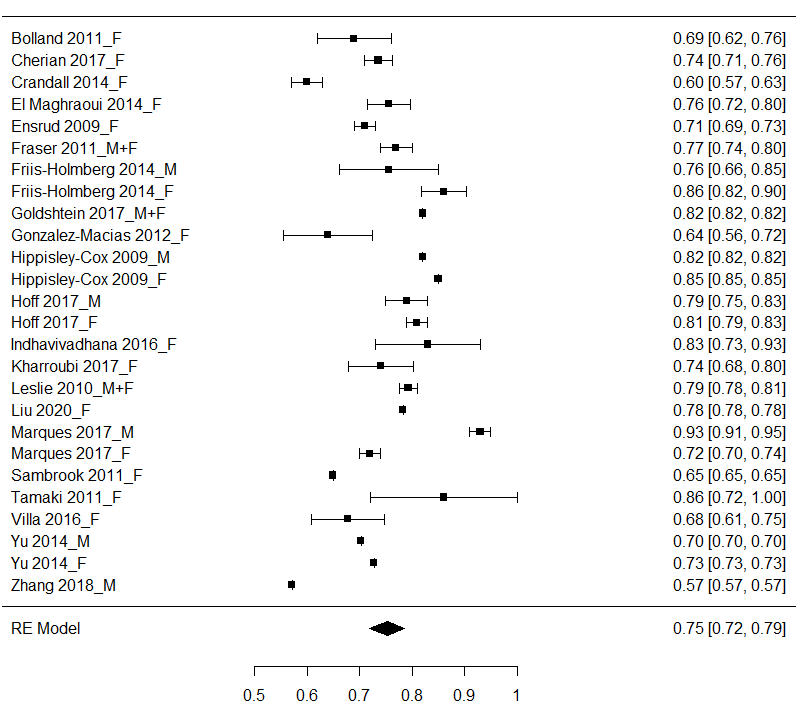


## Area under the curve, FRA-HS

### **WOMEN, MEN OR BOTH**

**MOF and HIP without BMD**


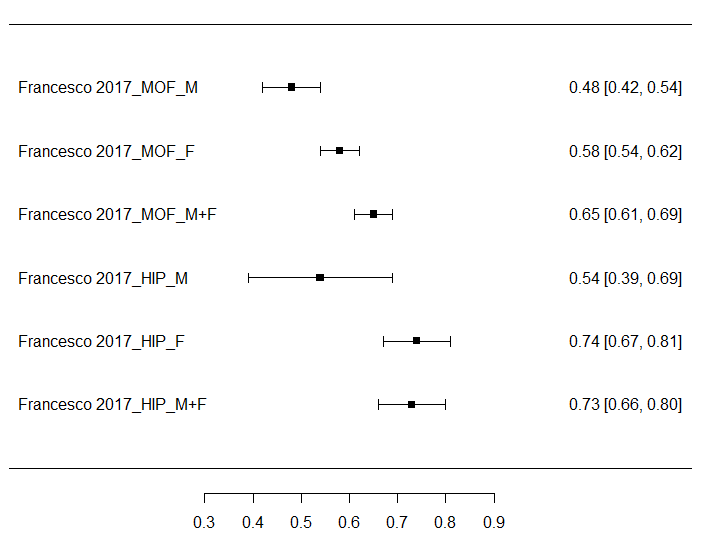


## Area under the curve, DeFRA and FRAX

### **WOMEN**

**MOF with BMD**


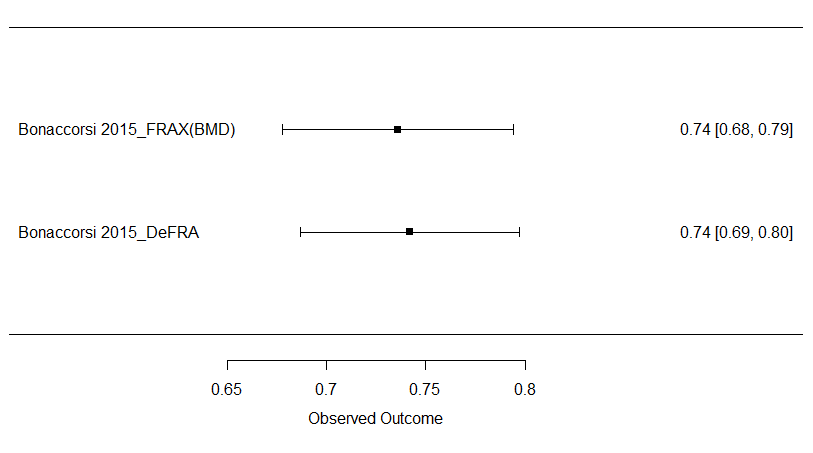


### **DIABETIC POPULATION**

**MOF with BMD**


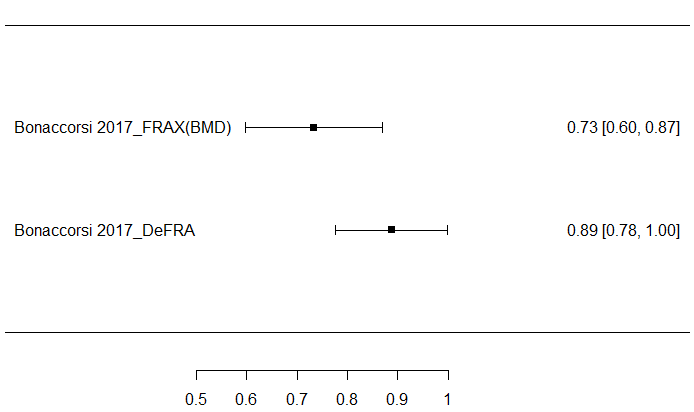


# Complete list of experts involved

**Fragility Fracture Team**

*Giovanni Adami Member of SIOMMMS - Società Italiana di Osteoporosi del metabolismo minerale e delle malattie dello scheletro*

*Rosaria Alvaro Associate Professor in Nursing Sciences – University of Rome Tor Vergata*

*Annalisa Biffi Department of Statistics and Quantitative Methods; Healthcare Research & Pharmacoepidemiology Interuniversity Center, University of Milan-Bicocca*

*Riccardo Bogini General Pratictioner at USL Umbria*

*Maria Luisa Brandi Full Professor of Endocrinology and Metabolic Bone Diseases - University of Florence; Director of the Regional Referral Center for Hereditary Endocrine Tumors; Director Clinical Unit on Metabolic Bone Disorders - University Hospital of Florence; President of FIRMO - Fondazione Italiana Ricerca sulle Malattie dell’Osso*

*Achille Patrizio Caputi Emeritus Professor of Pharmacology; University of Messina*

*Luisella Cianferotti Associate Professor of Endocrinology, University of Florence; member of FIRMO - Fondazione Italiana Ricerca sulle Malattie dell’Osso*

*Giovanni Corrao Full Professor of Medical Statistics – University of Milan-Bicocca; Director of Healthcare Research & Pharmacoepidemiology Interuniversity Center*

*Bruno Frediani Full Professor of Rheumatology; Director of the School of Specialization in Rheumatology - University of Siena; Director of the Complex Operational Unit in Rheumatology; Director of the Department of Medical Sciences*

*Davide Gatti Associate Professor of Rheumatology – University of Verona; President of the Scientific Committee of the ASITOI - Associazione Italiana Osteogenesi Imperfetta; Coordinator of the Guideline Commission SIOMMMS - Società Italiana di Osteoporosi del metabolismo minerale e delle malattie dello scheletro*

*Stefano Gonnelli Full Professor of Internal Medicine and Director of the School of Specialization in Iternal Medicine - University of Siena*

*Giovanni Iolascon Full Professor of Physical and Rehabilitation Medicine – University of Campania “Luigi Vanvitelli”*

*Andrea Lenzi Full Professor of Endocrinology - University of Rome La Sapienza; member of SIE – Società italiana di Endocrinologia*

*Salvatore Leone Member of AMICI Onlus - Associazione nazionale per le Malattie Infiammatorie Croniche dell'Intestino*

*Raffaella Michieli National Secretary SIMG – Società italiana di medicina generale e delle cure primarie*

*Silvia Migliaccio Member of SIE – Società italiana di Endocrinologia; Associate Professor - University of Rome Foro Italico*

*Tiziana Nicoletti Manager of CnAMC - Coordinamento nazionale delle Associazioni dei Malati Cronici e rari di Cittadinanzattiva*

*Marco Paoletta Member of SIMFER - Società Italiana di Medicina Fisica e Riabilitativa*

*Annalisa Pennini Member of FNOPI - Federazione Nazionale degli Ordini delle Professioni Infermieristiche per il progetto Fratture da Fragilità*

*Eleonora Piccirilli Department of Orthopedics and Traumatology, University of Rome Tor Vergata*

*Gloria Porcu Department of Statistics and Quantitative Methods; Healthcare Research & Pharmacoepidemiology Interuniversity Center, University of Milan-Bicocca*

*Raffaella Ronco Department of Statistics and Quantitative Methods; Healthcare Research & Pharmacoepidemiology Interuniversity Center, University of Milan-Bicocca*

*Maurizio Rossini Full Professor of Rheumatology; President of SIOMMMS - Società Italiana dell'Osteoporosi, del Metabolismo Minerale e delle Malattie dello Scheletro; Member of SIR - Società Italiana di Reumatologia*

*Umberto Tarantino Full Professor of Diseases of the Locomotor System – University of Rome; Member of SIOT – Società italiana di ortopedia e traumatologia*
