# Supplementary material for: A systematic review on the performance of fracture risk assessment tools: FRAX, DeFRA, FRA-HS
Source: J Endocrinol Invest. 2023 Apr 9;46(11):2287–97. doi: 10.1007/s40618-023-02082-8 (PMC10558377; doi:10.1007/s40618-023-02082-8)
Supplement: Supplementary file 3 — Supplementary file3 (DOCX 18 KB) [file 40618_2023_2082_MOESM3_ESM.docx]

**Supplemental Table S2.** Quality evaluation with the QUADAS-2

| *Study* | Risk of Bias | | | | Applicability concerns | | |
| --- | --- | --- | --- | --- | --- | --- | --- |
|  | Patient selection | Index test | Reference standard | Flow and timing | Patient selection | Index test | Reference standard |
| Bonaccorsi 2015 | Low | Low | Low | Low | Yes | Yes | Yes |
| Dagan 2017 | Low | Low | Low | Low | Yes | Yes | Yes |
| Fraser 2011 | Low | Low | Low | Low | Yes | Yes | Yes |
| Friis - Holmberg 2014 | Low | Low | Low | Low | Yes | Yes | Yes |
| Goldshtein 2018 | Low | Low | Low | Low | Yes | Yes | Yes |
| González-Macías 2012 | Low | Low | Low | Low | Yes | Yes | Yes |
| Hoff 2017 | Low | Low | Low | Low | Yes | Yes | Yes |
| Lapi 2017 | Low | Low | Low | Low | Yes | Yes | Yes |
| Leslie 2010 | Low | Low | Low | Low | Yes | Yes | Yes |
| Marques 2017 | Low | Low | Low | Low | Yes | Yes | Yes |
| Sandhu 2010 | High | Low | Low | Low | Unclear | Yes | Yes |
| Su 2017 | Unclear | Low | Low | Low | Yes | Yes | Yes |
| Tamaki 2011 | Low | Low | Low | Low | Yes | Yes | Yes |
| van Geel 2014 | Low | Low | Low | Low | Yes | Yes | Yes |
| Yu 2014 | Low | Low | Low | Low | Yes | Yes | Yes |
| Zhang 2018 | Low | Low | Low | Low | Yes | Yes | Yes |
| Pluskewicz 2014 | Unclear | Low | Low | Low | Unclear | Yes | Yes |
| Lin 2016 | High | Low | Low | Low | Unclear | Yes | Yes |
| Bonaccorsi 2017 | High | Low | Low | Low | Unclear | Yes | Yes |
| Czerwiński 2013 | Low | Low | Low | Low | Yes | Yes | Yes |
| Villa 2016 | Low | Low | Low | Low | Yes | Yes | Yes |
| Rubin 2013 | Unclear | Low | Low | Low | Yes | Yes | Yes |
| Bolland 2011 | Low | Low | Low | Low | Yes | Yes | Yes |
| Briot 2013 | Low | Low | Low | Low | Yes | Yes | Yes |
| Ensrud 2009 | Low | Low | Low | Low | Yes | Yes | Yes |
| Henry 2011 | Low | Low | Low | Low | Yes | Yes | Yes |
| Pluskiewicz 2010 | Low | Low | Low | Low | Yes | Yes | Yes |
| Sornay-Rendu 2010 | Low | Low | Low | Low | Yes | Yes | Yes |
| Trémollierres 2010 | Low | Low | Low | Low | Yes | Yes | Yes |
| Cherian 2018 | Unclear | Low | Low | Low | Yes | Yes | Yes |
| Kharroubi 2017 | Unclear | Low | Low | Low | Yes | Yes | Yes |
| Chandran 2018 | Low | Low | Low | Low | Yes | Yes | Yes |
| Kral 2017 | High | Low | Low | Low | Yes | Yes | Yes |
| Crandall 2014 | Unclear | Low | Low | Low | Yes | Yes | Yes |
| Cheung 2014 | Low | Low | Low | Low | Yes | Yes | Yes |
| Liu 2020 | Low | Low | Low | Low | Yes | Yes | Yes |
| El Maghraoui 2014 | Low | Low | Low | Low | Yes | Yes | Yes |
| Indhavivaghana 2016 | Unclear | Low | Low | Low | Yes | Yes | Yes |
| Tebé Cordomí 2013 | Low | Low | Low | Low | Yes | Yes | Yes |
| Hippisley – Cox 2009 | Low | Low | Low | Low | Yes | Yes | Yes |
| Sambrook 2011 | Low | Low | Low | Low | Yes | Yes | Yes |
| Cummins 2011 | High | Low | Low | Low | Unclear | Yes | Yes |
| Tanaka 2010 | Low | Low | Low | Low | Unclear | Yes | Yes |
