# Supplementary material for: A systematic review on the performance of fracture risk assessment tools: FRAX, DeFRA, FRA-HS
Source: J Endocrinol Invest. 2023 Apr 9;46(11):2287–97. doi: 10.1007/s40618-023-02082-8 (PMC10558377; doi:10.1007/s40618-023-02082-8)
Supplement: Supplementary file 4 — Supplementary file4 (DOCX 47 KB) [file 40618_2023_2082_MOESM4_ESM.docx]

**Supplemental Table S3.** Summary of Findings Tables.

**MOF: FRAX without BMD at 3% used to diagnose fracture in fracture-free or fractured patients**

| \| Sensitivity (median) \| 0.57 (95% CI: 0.49 to 0.64) \| \| --- \| --- \| \| Specificity (median) \| 0.69 (95% CI: 0.58 to 0.79) \| |  | \| Prevalences (median) \| 57% \|  \|  \| \| --- \| --- \| --- \| --- \| |  |
| --- | --- | --- | --- | --- | --- | --- | --- | --- | --- | --- | --- |

| Outcome | № of studies  (№ of patients) | Study design | Factors that may decrease certainty of evidence | | | | | Effect per 1.000 patients tested | Test accuracy CoE |
| --- | --- | --- | --- | --- | --- | --- | --- | --- | --- |
|  |  |  | Risk of bias | Indirectness | Inconsistency | Imprecision | Publication bias | pre-test probability of 57% |  |
| **True positives** (patients with fracture) | 3 studies 498 patients | cross-sectional (cohort type accuracy study) | not serious | not serious | serious ^a^ | serious ^b^ | none | 325 (279 to 365) | ⨁⨁◯◯ LOW |
| **False negatives** (patients incorrectly classified as not having fracture) |  |  |  |  |  |  |  | 245 (205 to 291) |  |
| **True negatives** (patients without fracture) | 3 studies 887 patients | cross-sectional (cohort type accuracy study) | not serious | not serious | serious ^a^ | very serious ^b^ | none | 297 (249 to 340) | ⨁◯◯◯ VERY LOW |
| **False positives** (patients incorrectly classified as having fracture) |  |  |  |  |  |  |  | 133 (90 to 181) |  |

Explanations

a. Studies were downgraded by one increment for inconsistency (was assessed by inspection of the sensitivity/specificity RevMan 5.4 plots).

b. Downgrading by one increment was applied for confidence intervals 10-20% or by two increments for confidence intervals more than 20%.

**MOF: FRAX without BMD at 5% used to diagnose fracture in fracture-free or fractured patients**

| \| Sensitivity (median) \| 0.34 (95% CI: 0.27 to 0.43) \| \| --- \| --- \| \| Specificity (median) \| 0.85 (95% CI: 0.79 to 0.90) \| |  | \| Prevalences (median) \| 38% \|  \|  \| \| --- \| --- \| --- \| --- \| |  |
| --- | --- | --- | --- | --- | --- | --- | --- | --- | --- | --- | --- |

| Outcome | № of studies  (№ of patients) | Study design | Factors that may decrease certainty of evidence | | | | | Effect per 1.000 patients tested | Test accuracy CoE |
| --- | --- | --- | --- | --- | --- | --- | --- | --- | --- |
|  |  |  | Risk of bias | Indirectness | Inconsistency | Imprecision | Publication bias | pre-test probability of 38% |  |
| **True positives** (patients with fracture) | 2 studies 287 patients | cross-sectional (cohort type accuracy study) | not serious | not serious | not serious | serious ^a^ | none | 131 (101 to 163) | ⨁⨁⨁◯ MODERATE |
| **False negatives** (patients incorrectly classified as not having fracture) |  |  |  |  |  |  |  | 249 (217 to 279) |  |
| **True negatives** (patients without fracture) | 2 studies 815 patients | cross-sectional (cohort type accuracy study) | not serious | not serious | not serious | serious ^a^ | none | 527 (487 to 558) | ⨁⨁⨁◯ MODERATE |
| **False positives** (patients incorrectly classified as having fracture) |  |  |  |  |  |  |  | 93 (62 to 133) |  |

Explanations

a. Downgrading by one increment was applied for confidence intervals 10-20% or by two increments for confidence intervals more than 20%.

**MOF: FRAX without BMD at 10% used to diagnose fracture in fracture-free or fractured patients**

| \| Sensitivity (median) \| 0.57 (95% CI: 0.52 to 0.64) \| \| --- \| --- \| \| Specificity (median) \| 0.66 (95% CI: 0.64 to 0.66) \| |  | \| Prevalences (median) \| 9% \|  \|  \| \| --- \| --- \| --- \| --- \| |  |
| --- | --- | --- | --- | --- | --- | --- | --- | --- | --- | --- | --- |

| Outcome | № of studies  (№ of patients) | Study design | Factors that may decrease certainty of evidence | | | | | Effect per 1.000 patients tested | Test accuracy CoE |
| --- | --- | --- | --- | --- | --- | --- | --- | --- | --- |
|  |  |  | Risk of bias | Indirectness | Inconsistency | Imprecision | Publication bias | pre-test probability of 9% |  |
| **True positives** (patients with fracture) | 6 studies 4602 patients | cross-sectional (cohort type accuracy study) | not serious | not serious | very serious ^a^ | serious ^b^ | none | 51 (47 to 58) | ⨁◯◯◯ VERY LOW |
| **False negatives** (patients incorrectly classified as not having fracture) |  |  |  |  |  |  |  | 39 (32 to 43) |  |
| **True negatives** (patients without fracture) | 6 studies 49855 patients | cross-sectional (cohort type accuracy study) | not serious | not serious | very serious ^a^ | not serious | none | 596 (582 to 601) | ⨁⨁◯◯ LOW |
| **False positives** (patients incorrectly classified as having fracture) |  |  |  |  |  |  |  | 314 (309 to 328) |  |

Explanations

a. Studies were downgraded by one increment for inconsistency (was assessed by inspection of the sensitivity/specificity RevMan 5.4 plots).

b. Downgrading by one increment was applied for confidence intervals 10-20% or by two increments for confidence intervals more than 20%.

**MOF: FRAX without BMD at 20% used to diagnose fracture in fracture-free or fractured patients**

| \| Sensitivity (median) \| 0.16 (95% CI: 0.13 to 0.20) \| \| --- \| --- \| \| Specificity (median) \| 0.93 (95% CI: 0.93 to 0.94) \| |  | \| Prevalences (median) \| 9% \|  \|  \| \| --- \| --- \| --- \| --- \| |  |
| --- | --- | --- | --- | --- | --- | --- | --- | --- | --- | --- | --- |

| Outcome | № of studies  (№ of patients) | Study design | Factors that may decrease certainty of evidence | | | | | Effect per 1.000 patients tested | Test accuracy CoE |
| --- | --- | --- | --- | --- | --- | --- | --- | --- | --- |
|  |  |  | Risk of bias | Indirectness | Inconsistency | Imprecision | Publication bias | pre-test probability of 9% |  |
| **True positives** (patients with fracture) | 3 studies 3226 patients | cross-sectional (cohort type accuracy study) | not serious | not serious | serious ^a^ | not serious | none | 14 (12 to 18) | ⨁⨁⨁◯ MODERATE |
| **False negatives** (patients incorrectly classified as not having fracture) |  |  |  |  |  |  |  | 76 (72 to 78) |  |
| **True negatives** (patients without fracture) | 3 studies 43580 patients | cross-sectional (cohort type accuracy study) | not serious | not serious | serious ^a^ | not serious | none | 846 (846 to 855) | ⨁⨁⨁◯ MODERATE |
| **False positives** (patients incorrectly classified as having fracture) |  |  |  |  |  |  |  | 64 (55 to 64) |  |

Explanations

a. Studies were downgraded by one increment for inconsistency (was assessed by inspection of the sensitivity/specificity RevMan 5.4 plots).

**MOF: FRAX without BMD at 30% used to diagnose fracture in fracture-free or fractured patients**

| \| Sensitivity (median) \| 0.04 (95% CI: 0.03 to 0.11) \| \| --- \| --- \| \| Specificity (median) \| 0.99 (95% CI: 0.98 to 0.99) \| |  | \| Prevalences (median) \| 9% \|  \|  \| \| --- \| --- \| --- \| --- \| |  |
| --- | --- | --- | --- | --- | --- | --- | --- | --- | --- | --- | --- |

| Outcome | № of studies  (№ of patients) | Study design | Factors that may decrease certainty of evidence | | | | | Effect per 1.000 patients tested | Test accuracy CoE |
| --- | --- | --- | --- | --- | --- | --- | --- | --- | --- |
|  |  |  | Risk of bias | Indirectness | Inconsistency | Imprecision | Publication bias | pre-test probability of 9% |  |
| **True positives** (patients with fracture) | 3 studies 3227 patients | cross-sectional (cohort type accuracy study) | not serious | not serious | not serious | not serious | none | 4 (3 to 10) | ⨁⨁⨁⨁ HIGH |
| **False negatives** (patients incorrectly classified as not having fracture) |  |  |  |  |  |  |  | 86 (80 to 87) |  |
| **True negatives** (patients without fracture) | 3 studies 43579 patients | cross-sectional (cohort type accuracy study) | not serious | not serious | not serious | not serious | none | 901 (892 to 901) | ⨁⨁⨁⨁ HIGH |
| **False positives** (patients incorrectly classified as having fracture) |  |  |  |  |  |  |  | 9 (9 to 18) |  |

**MOF: FRAX with BMD at 10% used to diagnose fracture in fracture-free or fractured patients**

| \| Sensitivity (median) \| 0.62 (95% CI: 0.52 to 0.70) \| \| --- \| --- \| \| Specificity (median) \| 0.73 (95% CI: 0.72 to 0.75) \| |  | \| Prevalences (median) \| 9% \|  \|  \| \| --- \| --- \| --- \| --- \| |  |
| --- | --- | --- | --- | --- | --- | --- | --- | --- | --- | --- | --- |

| Outcome | № of studies  (№ of patients) | Study design | Factors that may decrease certainty of evidence | | | | | Effect per 1.000 patients tested | Test accuracy CoE |
| --- | --- | --- | --- | --- | --- | --- | --- | --- | --- |
|  |  |  | Risk of bias | Indirectness | Inconsistency | Imprecision | Publication bias | pre-test probability of 9% |  |
| **True positives** (patients with fracture) | 7 studies 4706 patients | cross-sectional (cohort type accuracy study) | not serious | not serious | serious ^a^ | serious ^b^ | none | 56 (47 to 63) | ⨁⨁◯◯ LOW |
| **False negatives** (patients incorrectly classified as not having fracture) |  |  |  |  |  |  |  | 34 (27 to 43) |  |
| **True negatives** (patients without fracture) | 7 studies 52204 patients | cross-sectional (cohort type accuracy study) | not serious | not serious | very serious ^a^ | not serious | none | 664 (655 to 683) | ⨁⨁◯◯ LOW |
| **False positives** (patients incorrectly classified as having fracture) |  |  |  |  |  |  |  | 246 (227 to 255) |  |

Explanations

a. Studies were downgraded by one increment for inconsistency (was assessed by inspection of the sensitivity/specificity RevMan 5.4 plots).

b. Downgrading by one increment was applied for confidence intervals 10-20% or by two increments for confidence intervals more than 20%.

**MOF: FRAX with BMD at 20% used to diagnose fracture in fracture-free or fractured patients**

| \| Sensitivity (median) \| 0.19 (95% CI: 0.17 to 0.21) \| \| --- \| --- \| \| Specificity (median) \| 0.94 (95% CI: 0.93 to 0.94) \| |  | \| Prevalences (median) \| 10% \|  \|  \| \| --- \| --- \| --- \| --- \| |  |
| --- | --- | --- | --- | --- | --- | --- | --- | --- | --- | --- | --- |

| Outcome | № of studies  (№ of patients) | Study design | Factors that may decrease certainty of evidence | | | | | Effect per 1.000 patients tested | Test accuracy CoE |
| --- | --- | --- | --- | --- | --- | --- | --- | --- | --- |
|  |  |  | Risk of bias | Indirectness | Inconsistency | Imprecision | Publication bias | pre-test probability of 10% |  |
| **True positives** (patients with fracture) | 6 studies 10685 patients | cross-sectional (cohort type accuracy study) | not serious | not serious | not serious | not serious | none | 19 (17 to 21) | ⨁⨁⨁⨁ HIGH |
| **False negatives** (patients incorrectly classified as not having fracture) |  |  |  |  |  |  |  | 81 (79 to 83) |  |
| **True negatives** (patients without fracture) | 6 studies 98036 patients | cross-sectional (cohort type accuracy study) | not serious | not serious | not serious | not serious | none | 842 (833 to 842) | ⨁⨁⨁⨁ HIGH |
| **False positives** (patients incorrectly classified as having fracture) |  |  |  |  |  |  |  | 58 (58 to 67) |  |

**MOF: FRAX with BMD at 30% used to diagnose fracture in fracture-free or fractured patients**

| \| Sensitivity (median) \| 0.06 (95% CI: 0.04 to 0.08) \| \| --- \| --- \| \| Specificity (median) \| 0.98 (95% CI: 0.98 to 0.99) \| |  | \| Prevalences (median) \| 7.5% \|  \|  \| \| --- \| --- \| --- \| --- \| |  |
| --- | --- | --- | --- | --- | --- | --- | --- | --- | --- | --- | --- |

| Outcome | № of studies  (№ of patients) | Study design | Factors that may decrease certainty of evidence | | | | | Effect per 1.000 patients tested | Test accuracy CoE |
| --- | --- | --- | --- | --- | --- | --- | --- | --- | --- |
|  |  |  | Risk of bias | Indirectness | Inconsistency | Imprecision | Publication bias | pre-test probability of 7.5% |  |
| **True positives** (patients with fracture) | 3 studies 3178 patients | cross-sectional (cohort type accuracy study) | not serious | not serious | not serious | not serious | none | 5 (3 to 6) | ⨁⨁⨁⨁ HIGH |
| **False negatives** (patients incorrectly classified as not having fracture) |  |  |  |  |  |  |  | 70 (69 to 72) |  |
| **True negatives** (patients without fracture) | 3 studies 43628 patients | cross-sectional (cohort type accuracy study) | not serious | not serious | not serious | not serious | none | 907 (907 to 916) | ⨁⨁⨁⨁ HIGH |
| **False positives** (patients incorrectly classified as having fracture) |  |  |  |  |  |  |  | 18 (9 to 18) |  |

**HIP: FRAX without BMD at 3% used to diagnose fracture in fracture-free or fractured patients**

| \| Sensitivity (median) \| 0.59 (95% CI: 0.55 to 0.64) \| \| --- \| --- \| \| Specificity (median) \| 0.81 (95% CI: 0.79 to 0.82) \| |  | \| Prevalences (median) \| 1.5% \|  \|  \| \| --- \| --- \| --- \| --- \| |  |
| --- | --- | --- | --- | --- | --- | --- | --- | --- | --- | --- | --- |

| Outcome | № of studies  (№ of patients) | Study design | Factors that may decrease certainty of evidence | | | | | Effect per 1.000 patients tested | Test accuracy CoE |
| --- | --- | --- | --- | --- | --- | --- | --- | --- | --- |
|  |  |  | Risk of bias | Indirectness | Inconsistency | Imprecision | Publication bias | pre-test probability of 1.5% |  |
| **True positives** (patients with fracture) | 6 studies 8118 patients | cross-sectional (cohort type accuracy study) | not serious | not serious | very serious ^a^ | serious ^b^ | none | 9 (8 to 10) | ⨁◯◯◯ VERY LOW |
| **False negatives** (patients incorrectly classified as not having fracture) |  |  |  |  |  |  |  | 6 (5 to 7) |  |
| **True negatives** (patients without fracture) | 6 studies 916671 patients | cross-sectional (cohort type accuracy study) | not serious | not serious | very serious ^a^ | not serious | none | 803 (778 to 808) | ⨁⨁◯◯ LOW |
| **False positives** (patients incorrectly classified as having fracture) |  |  |  |  |  |  |  | 182 (177 to 207) |  |

Explanations

a. Studies were downgraded by one increment for inconsistency (was assessed by inspection of the sensitivity/specificity RevMan 5.4 plots).

b. Downgrading by one increment was applied for confidence intervals 10-20% or by two increments for confidence intervals more than 20%.

**HIP: FRAX without BMD at 5% used to diagnose fracture in fracture-free or fractured patients**

| \| Sensitivity (median) \| 0.47 (95% CI: 0.42 to 0.53) \| \| --- \| --- \| \| Specificity (median) \| 0.82 (95% CI: 0.82 to 0.83) \| |  | \| Prevalences (median) \| 4% \|  \|  \| \| --- \| --- \| --- \| --- \| |  |
| --- | --- | --- | --- | --- | --- | --- | --- | --- | --- | --- | --- |

| Outcome | № of studies  (№ of patients) | Study design | Factors that may decrease certainty of evidence | | | | | Effect per 1.000 patients tested | Test accuracy CoE |
| --- | --- | --- | --- | --- | --- | --- | --- | --- | --- |
|  |  |  | Risk of bias | Indirectness | Inconsistency | Imprecision | Publication bias | pre-test probability of 4% |  |
| **True positives** (patients with fracture) | 6 studies 8497 patients | cross-sectional (cohort type accuracy study) | not serious | not serious | very serious ^a^ | serious ^b^ | none | 19 (17 to 21) | ⨁◯◯◯ VERY LOW |
| **False negatives** (patients incorrectly classified as not having fracture) |  |  |  |  |  |  |  | 21 (19 to 23) |  |
| **True negatives** (patients without fracture) | 6 studies 924487 patients | cross-sectional (cohort type accuracy study) | not serious | not serious | very serious ^a^ | not serious | none | 787 (787 to 797) | ⨁⨁◯◯ LOW |
| **False positives** (patients incorrectly classified as having fracture) |  |  |  |  |  |  |  | 173 (163 to 173) |  |

Explanations

a. Studies were downgraded by one increment for inconsistency (was assessed by inspection of the sensitivity/specificity RevMan 5.4 plots).

b. Downgrading by one increment was applied for confidence intervals 10-20% or by two increments for confidence intervals more than 20%.

**HIP: FRAX with BMD at 3% used to diagnose fracture in fracture-free or fractured patients**

| \| Sensitivity (median) \| 0.57 (95% CI: 0.50 to 0.63) \| \| --- \| --- \| \| Specificity (median) \| 0.80 (95% CI: 0.79 to 0.80) \| |  | \| Prevalences (median) \| 6% \|  \|  \| \| --- \| --- \| --- \| --- \| |  |
| --- | --- | --- | --- | --- | --- | --- | --- | --- | --- | --- | --- |

| Outcome | № of studies  (№ of patients) | Study design | Factors that may decrease certainty of evidence | | | | | Effect per 1.000 patients tested | Test accuracy CoE |
| --- | --- | --- | --- | --- | --- | --- | --- | --- | --- |
|  |  |  | Risk of bias | Indirectness | Inconsistency | Imprecision | Publication bias | pre-test probability of 6% |  |
| **True positives** (patients with fracture) | 6 studies 3045 patients | cross-sectional (cohort type accuracy study) | not serious | not serious | not serious | serious ^a^ | none | 34 (30 to 38) | ⨁⨁⨁◯ MODERATE |
| **False negatives** (patients incorrectly classified as not having fracture) |  |  |  |  |  |  |  | 26 (22 to 30) |  |
| **True negatives** (patients without fracture) | 6 studies 100431 patients | cross-sectional (cohort type accuracy study) | not serious | not serious | not serious | not serious | none | 747 (743 to 752) | ⨁⨁⨁⨁ HIGH |
| **False positives** (patients incorrectly classified as having fracture) |  |  |  |  |  |  |  | 193 (188 to 197) |  |

Explanations

a. Downgrading by one increment was applied for confidence intervals 10-20% or by two increments for confidence intervals more than 20%.

**HIP: FRAX with BMD at 5% used to diagnose fracture in fracture-free or fractured patients**

| \| Sensitivity (median) \| 0.43 (95% CI: 0.35 to 0.51) \| \| --- \| --- \| \| Specificity (median) \| 0.86 (95% CI: 0.84 to 0.88) \| |  | \| Prevalences (median) \| 6% \|  \|  \| \| --- \| --- \| --- \| --- \| |  |
| --- | --- | --- | --- | --- | --- | --- | --- | --- | --- | --- | --- |

| Outcome | № of studies  (№ of patients) | Study design | Factors that may decrease certainty of evidence | | | | | Effect per 1.000 patients tested | Test accuracy CoE |
| --- | --- | --- | --- | --- | --- | --- | --- | --- | --- |
|  |  |  | Risk of bias | Indirectness | Inconsistency | Imprecision | Publication bias | pre-test probability of 6% |  |
| **True positives** (patients with fracture) | 5 studies 1372 patients | cross-sectional (cohort type accuracy study) | not serious | not serious | serious ^a^ | serious ^b^ | none | 26 (21 to 31) | ⨁⨁◯◯ LOW |
| **False negatives** (patients incorrectly classified as not having fracture) |  |  |  |  |  |  |  | 34 (29 to 39) |  |
| **True negatives** (patients without fracture) | 5 studies 53089 patients | cross-sectional (cohort type accuracy study) | not serious | not serious | serious ^a^ | not serious | none | 808 (790 to 827) | ⨁⨁⨁◯ MODERATE |
| **False positives** (patients incorrectly classified as having fracture) |  |  |  |  |  |  |  | 132 (113 to 150) |  |

Explanations

a. Studies were downgraded by one increment for inconsistency (was assessed by inspection of the sensitivity/specificity RevMan 5.4 plots).

b. Downgrading by one increment was applied for confidence intervals 10-20% or by two increments for confidence intervals more than 20%.
